# The fitness landscape of a form II rubisco in a photosynthetic bacterium guides engineering of oxygen tolerance

**DOI:** 10.1126/sciadv.aee9222

**Published:** 2026-09-04

**Authors:** Ute A. Hoffmann, Axel F. Knave, Anna Z. Schuppe, Sebastià Capó-Bauçà, Pere Aguiló Nicolau, Karen Schriever, Anna Karlsson, Michele Russo, Emil Sporre, Mami Matsuda, Rui Miao, Tomohisa Hasunuma, Jeroni Galmés, Per-Olof Syrén, Elton P. Hudson

**Affiliations:** ^1^ School of Engineering Sciences in Chemistry, Biotechnology and Health, Science for Life Laboratory, KTH - Royal Institute of Technology; Stockholm, Sweden.; ^2^ Research Group on Plant Biology under Mediterranean Conditions, Universitat de les Illes Balears – INAGEA, Palma, Balearic Islands, Spain.; ^3^ Graduate School of Science, Technology and Innovation, Kobe University, Kobe, Japan.

## Abstract

Ribulose-1,5-bisphosphate carboxylase/oxygenase (rubisco) is an important but challenging protein engineering target. Fast and selective rubiscos could enhance photosynthesis in plants and accelerate biobased production processes. To facilitate engineering of rubisco, we applied an in vivo screen that couples rubisco activity to growth rate of the photoautotrophic cyanobacterium *Synechocystis sp.* PCC 6803. We screened a barcoded mutagenesis library of the form II rubisco from *Gallionella sp.* containing 15,000 single-site and multi-site variants. Exchanges in loop 6 near the active site, at the dimer interface, and in potential gas tunnels improved rubisco fitness. The dataset also informed protein engineering, using recombination and a trained transformer model. In vitro characterisation of two high-fitness variants showed reduced catalytic efficiency for oxygenation (*k*_cat_/K_o_) in both and an increased carboxylation turnover (*k*_cat_^C^) in one. This large labeled fitness dataset, containing examples of epistasis, can be useful for benchmarking computational models of rubisco.

## INTRODUCTION

Ribulose-1,5-bisphosphate carboxylase/oxygenase (rubisco) is an important engineering target for improving photosynthetic efficiency in plants (*1*, *2*). Enhancing the specific activity of rubisco could reduce nitrogen demand, while enhancing the selectivity of rubisco for CO_2_ over O_2_ could reduce the accumulation of 2-phosphoglycolate (2-PG), a byproduct that inhibits carbon fixation and must be salvaged by photorespiration (*3*, *4*). Rubisco activity may also limit carbon conversion in certain microbial bioproduction platforms, such as cyanobacteria and lithoautotrophic bacteria (*5*). However, protein engineering of rubisco is challenging. Form I plant rubiscos are hexadecameric, with both large and small subunits, and expression of these in bacteria requires multiple maturation factors and chaperones (*6*). Further, the observed correlation between carboxylation and oxygenation catalytic efficiencies in rubiscos (*7*, *8*) may be indicative of a mechanistic coupling (*9*), though it was proposed that plant rubiscos are evolving to increase carboxylation efficiency and selectivity (*10*, *11*). Bacterial rubiscos have been pursued as replacements for the plant enzyme in crops, as these are expressed at higher titers, and generally display higher carboxylation rates. However, the lower selectivity of bacterial rubiscos necessitates a high CO_2_ cultivation for the resultant transgenic plants (*12*–*16*).

There has been some success in enhancing the kinetic parameters of form I rubiscos from bacteria, such as increasing carbon fixation rate or reducing oxygen sensitivity, though rarely were these two achieved simultaneously (*17*–*20*). Form II rubiscos represent an alternative rubisco evolutionary lineage, primarily adapted to low O_2_ environments, and include the highest carboxylation rates reported (*21*–*23*). The simple format of form II rubiscos, which are homomultimeric and lack small subunits (*24*), may increase the available space for mutation since conservation of contacts to other proteins are not required (*25*–*27*). Form II rubiscos have also been subjected to extensive mutagenesis in order to map the determinants of speed and selectivity (*28*–*30*). This literature suggests that in some cases kinetic parameters can be modulated independently. The carboxylation rate of form II rubisco from *Riftia pachyptila* could be increased without affecting binding affinity (*31*). The O_2_ affinity of form II rubisco from *Gallionella* could be reduced with little effect on the carboxylation efficiency, resulting in rubisco mutants that had higher catalytic efficiency in air than the wild type (*22*). Form II rubiscos with high carboxylation efficiencies and reduced sensitivity to oxygen could be starting points for eventual transfer to plant or other bioproduction systems.

In vivo screening platforms for rubisco activity expand the number of variants that can be tested simultaneously. Such platforms can also be adapted to assay specific kinetic parameters. For example, changes in rubisco specificity factor (*S*_C/O_) can be estimated by comparing enrichment of mutant clones during oxic or anoxic growth, or CO_2_ affinity by growth at low CO_2_. To date, rubisco-dependent *E. coli* screens have relied on error-prone PCR (Ep-PCR) to generate enzyme diversity, and coupled these libraries to directed evolution schemes to enrich high-fitness variants that impart faster cell growth (*32*). Ep-PCR generates multi-site mutants, which allows study of epistatic relationships in a protein (*25*, *33*–*35*). However, Ep-PCR results in a large number of inactive enzymes. Furthermore, much of the information on enzyme fitness is lost, as less fit variants are not retained or characterized further. To limit the number of non-functional variants, input libraries can be designed using prior knowledge, such as phylogenetic relationships (*36*). Using prior knowledge has a higher likelihood of identifying a more fit variant than random input (*36*, *37*). To retain information on low-fitness variants, barcoding and tracking using deep sequencing is employed (*38*). Labeled fitness data, where enzyme sequences are matched to a phenotype, often consisting of thousands of data points, can be used to train machine-learning models to eventually guide precise engineering for enzyme catalytic parameters (*39*).

Recently, we established a rubisco screening strain of the cyanobacterium *Synechocystis* sp. PCC 6803 and demonstrated that the platform could be used to screen activity of the form II rubisco from *Gallionella* sp., CbbM (*40*). A cyanobacteria screening platform provides a closer evolutionary relationship to plants than non-photosynthetic bacteria, and may allow us to enrich rubiscos with properties that are relevant for photoautotrophy, such as improved resistance to in situ generated oxygen, or resistance to inhibition from prevalent sugar phosphates. Furthermore, faster rubiscos could be leveraged for promoting productivity of cyanobacteria in bioprocesses (*41*, *42*). We also established a combination of the phylogenetically-guided method EVmutation (*43*, *44*) and in silico evolution (*45*) to design higher-order rubisco variants to be tested in the cyanobacteria screening strain (*40*). Here, we extended the design approach by using both EVmutation and DeepSequence (*46*) to predict fitness values of variants identified by in silico evolution. We identified seven amino acid positions and specific exchanges at these positions, which had, according to both algorithms, a high likelihood to increase rubisco fitness. We then generated a large combinatorial rubisco library consisting of saturation/DMS and combinatorial parts exceeding 17,000 members. The library was introduced into the screening strain, and we performed growth competition experiments under different gas feed and light conditions. This resulted in an extended fitness landscape of *Gallionella* rubisco and was used to create second-generation rubisco variants. These variants were created both through a naive approach and by using the machine learning algorithm ProteinNPT (*47*, *48*). In addition to identifying new epistasis in rubisco and potential determinants of rubisco oxygen reactivity, this work provides a rubisco benchmark of fitness predictors and protein language models, as well as combinatorial labeled fitness dataset that will be useful for future training.

## RESULTS

**Fig. 1.**
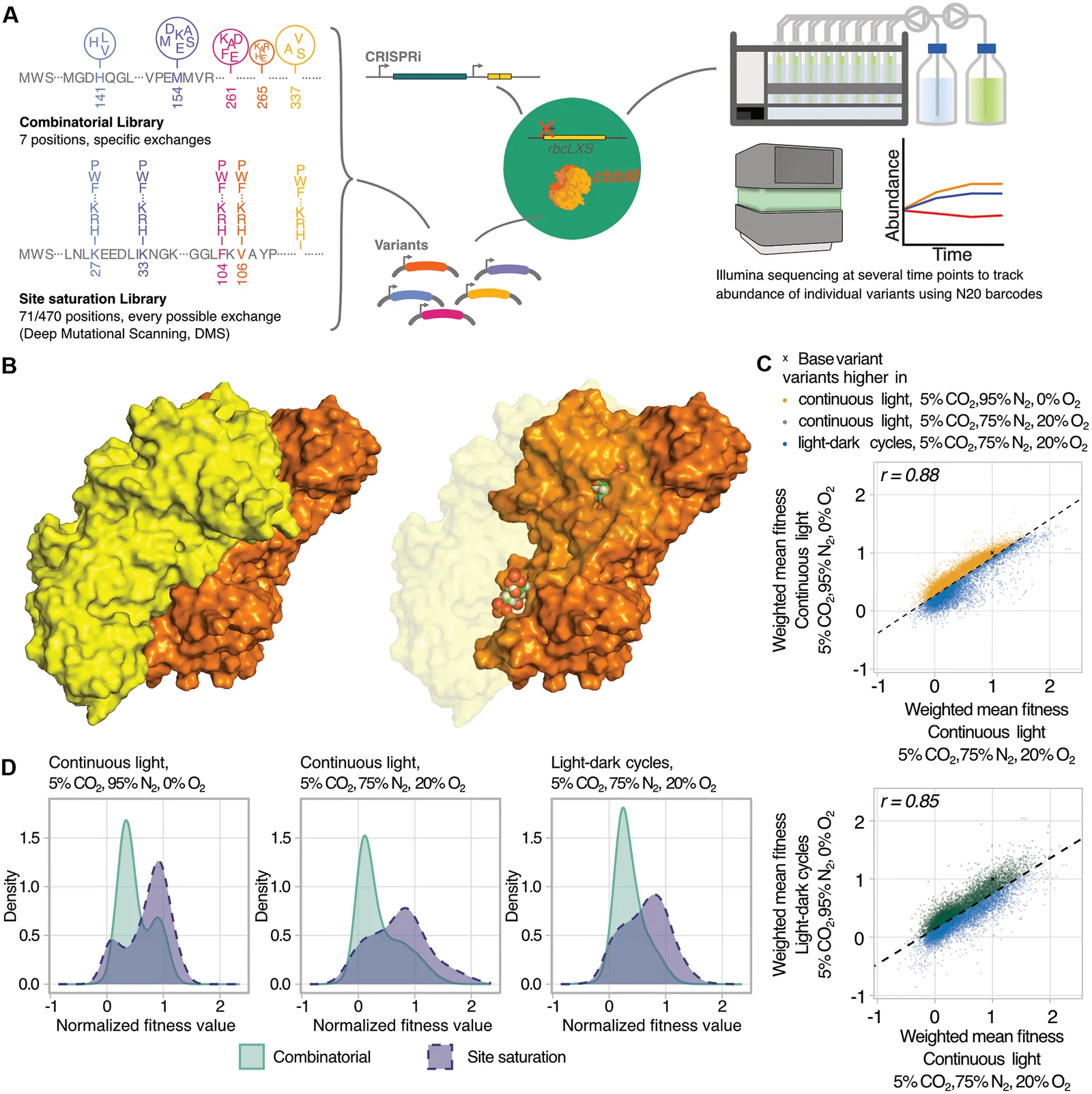
Workflow and fitness distributions of the CbbM library. (**A**). A combinatorial library with selected exchanges at seven positions and a saturational library with single amino acid exchanges were combined and introduced into a *Synechocystis* mutant strain in which the endogenous rubisco can be repressed by CRISPR inhibition (CRISPRi). The pooled *Synechocystis* was subjected to growth competition in three conditions. White light intensity was 300 μE in all cases. The abundance of individual mutant variants was tracked by Illumina sequencing of assigned 20-nucleotide barcodes. (**B**) Predicted structure of the CbbM WT dimeric enzyme with the CAP (an analog of RuBP) at the active site shown as spheres. (**C**) Comparison of normalized weighted mean fitness values for the complete library at different growth conditions. The dashed line gives the line defined by the linear regression of both conditions. Pearson’s r for the correlation of the respective conditions is 0.88 and 0.85, respectively. (**D**) Density plots of fitness distributions of combinatorial and saturation library parts at different growth conditions. Fitness values were normalized so that the CbbM_base variant had a fitness score of 1.0 and the median of fitness values for inactive K200 variants had a fitness score of 0.0.

We reported an in vivo screening platform for the heterologous rubisco CbbM in the photoautotrophic cyanobacterium *Synechocystis* sp. PCC 6803. (*40*). The native form I rubisco is repressed 95–99% using inducible CRISPR interference of the native *rbcLSX* operon. The growth of strain CRISPRi*_rbcLSX_* becomes dependent on the activity of the heterologous CbbM (Fig. 1A). As CbbM is not encapsulated in the *Synechocystis* carboxysomes, growth rate is also influenced by the CO_2_ level and the CO_2_:O_2_ ratio in the culture gas feed. We reasoned that this screening platform would make it possible to rank mutant rubiscos based on kinetic parameters such as k_cat_^c^, K_C_, and *S*_C/O_. In this work, we extended screening to a large, barcoded library of CbbM variants (Fig. 1A). The starting point was variant CbbM_base, a 2-point mutant of CbbM (S162P, G422R) that we previously found to impart a similar growth to *Synechocystis* as CbbM, but is more thermostable (CbbM_base T_m_ 50°C, CbbM T_m_ 44°C) (*40*). This variant was chosen as base for the library since thermostable enzymes tend to tolerate mutations to a higher degree (*49*).

### A phylogenetically-guided approach to designing an input library for high-throughput screening

We designed a CbbM_base library comprising both saturation and combinatorial mutagenesis of selected residues. Positions for saturation mutagenesis (totaling 71 positions) were chosen using phylogenetically-guided methods DeepSequence (*46*) and EVmutation (*43*, *44*), which use multiple sequence alignments (MSA) to identify residues of high conservation or that may be co-evolving. We selected residue positions for which the EVmutation epistatic score was above 1.0, indicating that exchange at this position could have a positive effect, or which were among the top 100 highest fitness predictions of DeepSequence and also had an EVmutation epistatic score above −1.0 (table S1). Additionally, we included 11 lysine residues for saturation mutagenesis. Non-specific acetylation of lysine can affect enzyme activity in bacteria (*50*), though there are no reports of this modification on CbbM. We also included N389 and N401, positions where mutation was previously shown to have a positive effect on expressibility or catalytic efficiency in rubisco homologues (*33*, *51*). Saturation mutagenesis of these 71 positions (15% of CbbM sequence) resulted in 1,349 variants (table S2; data file S1).

Positions for combinatorial mutagenesis were selected by coupling the output of EVmutation and DeepSequence to iterative rounds of in silico optimization, as described previously (*45*). This analysis identifies subsets of residues in CbbM and samples multiple amino acid exchanges at these residues to find combinations that are potentially fitness enhancing (*40*). Based on this, we selected 7 residues to be mutagenized with a reduced set of amino acid exchanges (Table 1). As expected from a phylogeny-based method, many of the suggested exchanges corresponded to amino acids present in rubisco homologues (fig. S1). The reduced set of exchanges were combined in every possible manner, yielding 16,200 possible variants, of which 16,178 contained more than one amino acid exchange (table S2). To enable tracking of different protein variants by Illumina sequencing, each gene variant was labeled with a N20 barcode. The CbbM saturation and combinatorial libraries were synthesized by commercial supplier (GeneScript). After transformation of the CbbM library into *E. coli,* barcodes were mapped to variant sequences using PacBio long-read sequencing. The *E. coli* plasmid pool contained >95% of the designed library variants. The plasmid pool was transformed into the *Synechocystis* screening strain. Deep sequencing of the *Synechocystis* pool showed an average of 2–3 barcodes per variant.

**Table 1.** Amino acid positions and possible replacement residues chosen for the CbbM combinatorial library.

Residue	Possible exchanges	Corresponding *R. rubrum* residue	Corresponding *Gallionella GWSB1* residue
H127	H, V, L	V118	I118
M140	M, E, D, K, S, A	A131	R131
K247	K, D, A, F, E	F238	Y238
H251	H, K, A, R, E	A242	A242
V323	V, A, S	A312	S312
Q392	Q, E, D	E381	E381
S432	S, A, I, T	V421	A421

### Single-site exchange library contains many rubisco variants with higher fitness

The resultant *Synechocystis* library was pooled and cultivated under three growth conditions in a multicultivator operating in turbidostat mode: continuous light with an oxygen-free gas feed of 5% CO_2_, 95% N_2_, 0% O_2_, continuous light and an oxygen-containing gas feed of 5% CO_2_, 70% N_2_, 20% O_2_, and light-dark cycles in the oxygen-containing gas feed. The average growth rate was dependent on the oxygen content (0.041 h^−1^ in 0% O_2_ condition and 0.028 h^−1^ in 20% O_2_ condition), and was significantly slower than wild type *Synechocystis* [0.08 h^−1^ in similar conditions (*40*, *52*)]. We note that due to in situ generated O_2_ during photosynthesis, the 0% O_2_ growth condition was not anoxic. Samples were periodically withdrawn from the bioreactors over 12 generations, and barcode frequency in the population quantified using Illumina sequencing (fig. S2). We verified RbcL repression during library cultivation by quantitative proteomics (fig. S3, table S3).

For each CbbM variant, we calculated a weighted mean-normalized fitness score, which accounted for all barcodes for a variant (fig. S4). Barcode sampling bias was mitigated by integrating data from the different time points at which samples were taken and by downweighting barcodes that had a high standard error or barcodes which did not correlate well with other barcodes from the same variant. Fitness scores were normalized for each condition so that fitness of the CbbM_base variant corresponded to 1, and the median fitness of all non-active K200 variants (*7*) corresponded to 0 (fig. S5). Calculated fitness values showed a bimodal distribution (Fig. 1D), implying a selection based on CbbM properties. The fraction of variants that had higher (*P* < 0.05 Wilcoxon Rank test) fitness than CbbM_base was 8% and 0.8% for the saturation and combinatorial libraries, respectively (table S4). Statistical significance depends on both effect size and the number of unique barcodes connected to a variant (fig. S4C).

Fitness scores were negatively correlated with residue conservation score (average Pearson’s r = −0.52; fig. S6), though there were non-conserved residues for which all replacements had a detrimental effect (G397, N398, and N401), and a few highly conserved residues were surprisingly tolerant to exchanges (I411, W114). A recent deep mutational scan of *Rhodospirillum rubrum* rubisco reported that solvent exposed residues were more tolerant to mutation than buried residues (*29*). The fitness of CbbM variants were moderately correlated (average Pearson’s r = 0.47; fig. S6) with solvent accessibility. Fitness at 21% and 0% O_2_ growth conditions strongly correlated (Fig. 1C, Pearson’s r > 0.8), though fitness increases were higher in the 21% O_2_ growth condition. Fitness scores of CbbM variants were also highly correlated between constant light and light-dark growth (Fig. 1C). In plants, multiple sugar phosphates have been shown to inhibit rubisco activity (*53*). However, rubisco inactivation in the dark in *Synechocystis* was recently shown to be regulated by RuBP availability and not enzyme inactivation by metabolites (*54*).

**Fig. 2.**
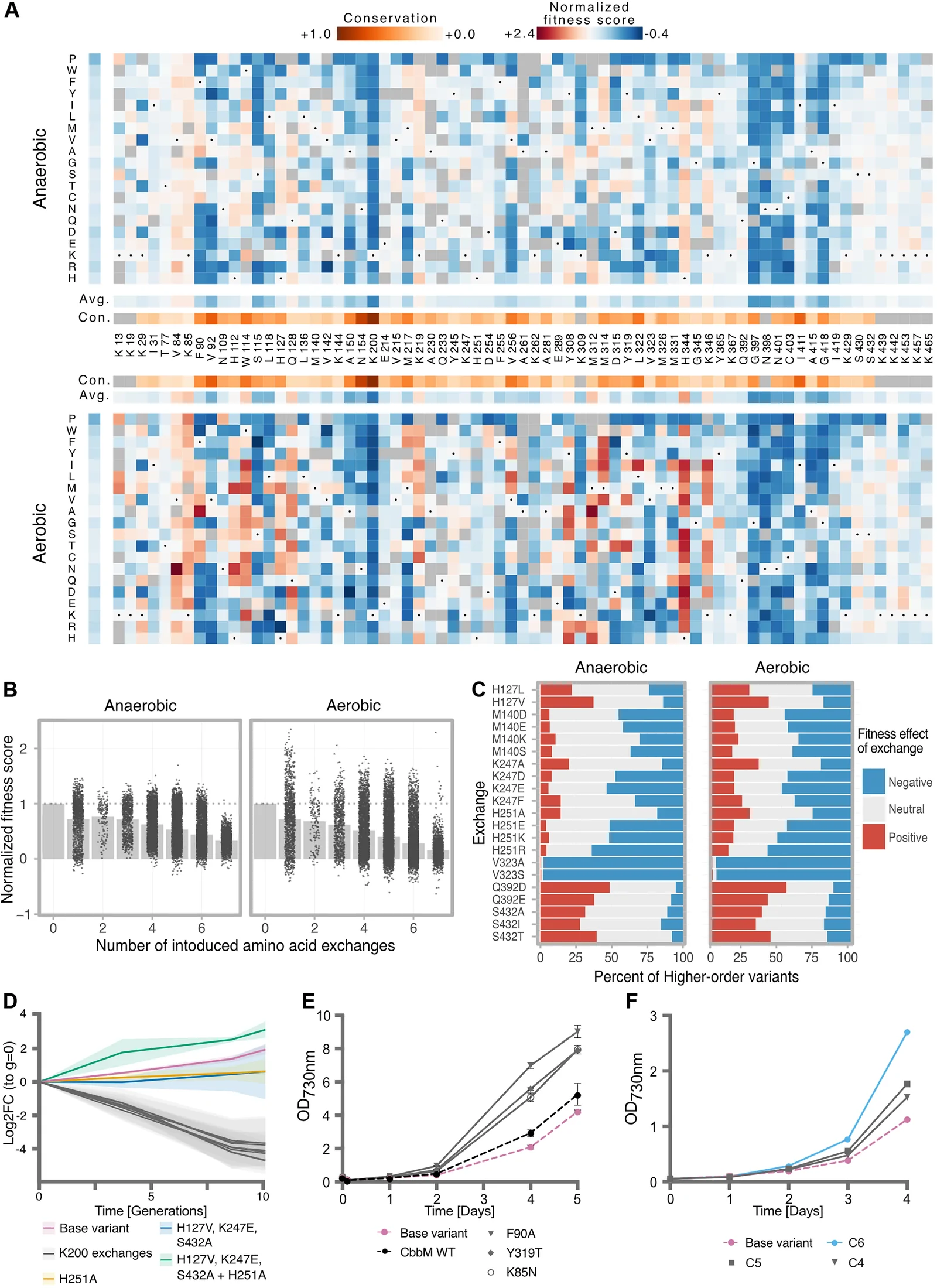
Fitness of single-site and combinatorial CbbM variants. (**A**) Heatmap of variant fitness of the saturation part of the library in two growth conditions. Values are normalized so that neutral mutations have value = 1 and inactive K200 variants have value 0. Gas conditions were 5% CO_2_, 95% N_2_, 0% O_2_ (annotated as “Anaerobic”), and 5% CO_2_, 75% N_2_, 20% O_2_ (annotated as “Aerobic”). Wild-type residues are indicated with a black dot. Gray squares indicate that there is no data available, either because the exchange was not covered by the MSA or because the variant dropped out of the library during construction. The conservation field is annotated with “Con.” The row annotated with “Avg” shows the average effect of all fitness scores shown in the plot in that row or column. (**B**) Normalized fitness scores of the combinatorial library variants plotted against the number of introduced mutations, in nitrogen cultivation and in air condition. Points are fitness scores for variants and bars are averages. (**C**) Global overview of fitness effects of specific amino acid exchanges in either saturation or combinatorial library in condition 5% CO_2_, 75% N_2_, 20% O_2_ . Data for all growth conditions in table S4. (**D**) Example of epistasis effect observed for H251A. (**E**) Batch cultivations of selected *Synechocystis* strains from the saturation library. Starting OD_730_ was 0.1 and conditions were 5% CO_2_, 75% N_2_, 20% O_2_ and 50 μE. Cultivations were performed in 6-well plates. (**F**) Batch cultivations of selected *Synechocystis* strains from the combinatorial library. Starting OD_730_ was 0.05 and conditions were 5% CO_2_, 75% N_2_, 20% O_2_ and 50 μE. Cultivations were performed in 6-well plates.

Replacements at a few positions had, on average, a positive effect on the normalized fitness value. Notable were residues H344 and K346, located within the C-terminal hinge of loop 6 which is proposed to be involved in CO_2_/O_2_ discrimination and catalysis (*30*, *55*–*57*). Most exchanges at H344 had a positive effect on fitness, except H344G and H344P, which had a negative effect (Fig. 2A). It is tempting to assume that these latter exchanges destabilize the loop by affecting backbone flexibility. Interestingly, exchanges H344W and H344Y were neutral, implying that the aromatic pi system of H344 may play a role at this position. Exchanges at D315, which may form a hydrogen bond to K346 to stabilize loop 6, were beneficial in cases where a hydrogen bond could be retained (D315E, D315S).

Residues in a stretch between G397 and I411 exhibited a consistently strong negative effect upon substitution under all conditions. This stretch forms an α-helix positioned above an opening that connects the cavity where RuBP binds to the protein surface (fig. S7A). The very low tolerance to mutations in these residues likely reflects the importance of maintaining this structural opening. Loss or alteration of this tunnel could prevent the enzyme from properly binding or releasing RuBP, 3PGA, and 2PG.

CbbM contains a nine residue insert (positions 78–87) that is not present in well-investigated form II rubiscos from *R. rubrum* and *Gallionella GW1SB* or in form I rubiscos from spinach and cyanobacteria. Structural predictions of the entire dimeric protein using both homology modeling and AlphaFold showed that this insert is in a loop region. Mutations at V84 and K85 within this insert generally increased fitness. Other positions at which, on average, replacements had a positive effect were Q128 and K219, which are part of a beta-sheet in the N-terminal domain and an alpha helix in the C-terminal domain, respectively.

Residue W114 is at the dimer-dimer interface, an area presumed to be less tolerant to mutation. However, mutations at W114 are among the highest fitness variants in the saturation library, and the effect was stronger in the aerobic than in the anaerobic condition. Tunnel analysis using CAVER 2.0 revealed that residue W114 lies adjacent to a continuous cavity or tunnel that extends through the enzyme and connects the two active sites of the dimer (fig. S7, C and D).

### Combinatorial mutagenesis reveals extensive epistatic interactions in rubisco

The average fitness of CbbM decreased with the number of introduced mutations (Fig. 2B), as reported for other proteins (*47*). While we found several higher-order variants with fitness exceeding that of single-site variants, the extent of such fitness increase was not high, suggesting that the zero-shot predictions for multi-site exchanges are significantly hindered by unseen epistatic effects. An illustrative example of epistasis is the three-point variant (H127V, K247E, S432A), and the single-point variant H251A, which both showed negative fitness compared to CbbM_base. However, the combined four-point variant showed significantly higher fitness (Fig. 2D).

For each of the 21 exchanges in the combinatorial library, we calculated the additive fitness of adding that exchange to a variant. Most exchanges could be either beneficial or detrimental, though there were some exceptions. Exchanges at V323 (V323A and V323S) were detrimental in all variants (Fig. 2C). The residue V323 appears to contact an extended tunnel which connects the protein surface to the active sites (fig. S7B), and the negative impact of substitutions suggests that this tunnel is essential for catalysis in CbbM_base. By contrast, mutations at surface residues H127, Q392, and S432 were rarely detrimental and frequently beneficial.

To validate results from the high-throughput fitness screen, we introduced several CbbM variants into the *Synechocystis* CRISPRi_rbcLSX_ strain and measured growth in batch cultivations. This validation included variants with high fitness values from the saturation mutagenesis library; variants K85N, F90A, and Y319T and combinatorial library; C4 (H127V, K247E, H251A, S432A), C5 (H127V, K247A, H251K, Q392E, S432I) and C6 (H127V, M140A, K247A, H251A, Q392E, S432T). All of the resultant *Synechocystis* strains showed faster growth than CbbM_base and CbbM_wt strains, in agreement with fitness data from library screening (Fig. 2, E and F). To determine whether the improved fitness was due to altered kinetic parameters or variant solubility (*58*), we compared CbbM abundances with western blotting and quantification by mass-spectrometry proteomics (fig. S8). Differences in abundances of the CbbM variants were minor.

### Zero-shot models predict fitness of single amino acid exchange mutants but not multi-site mutants

**Fig. 3.**
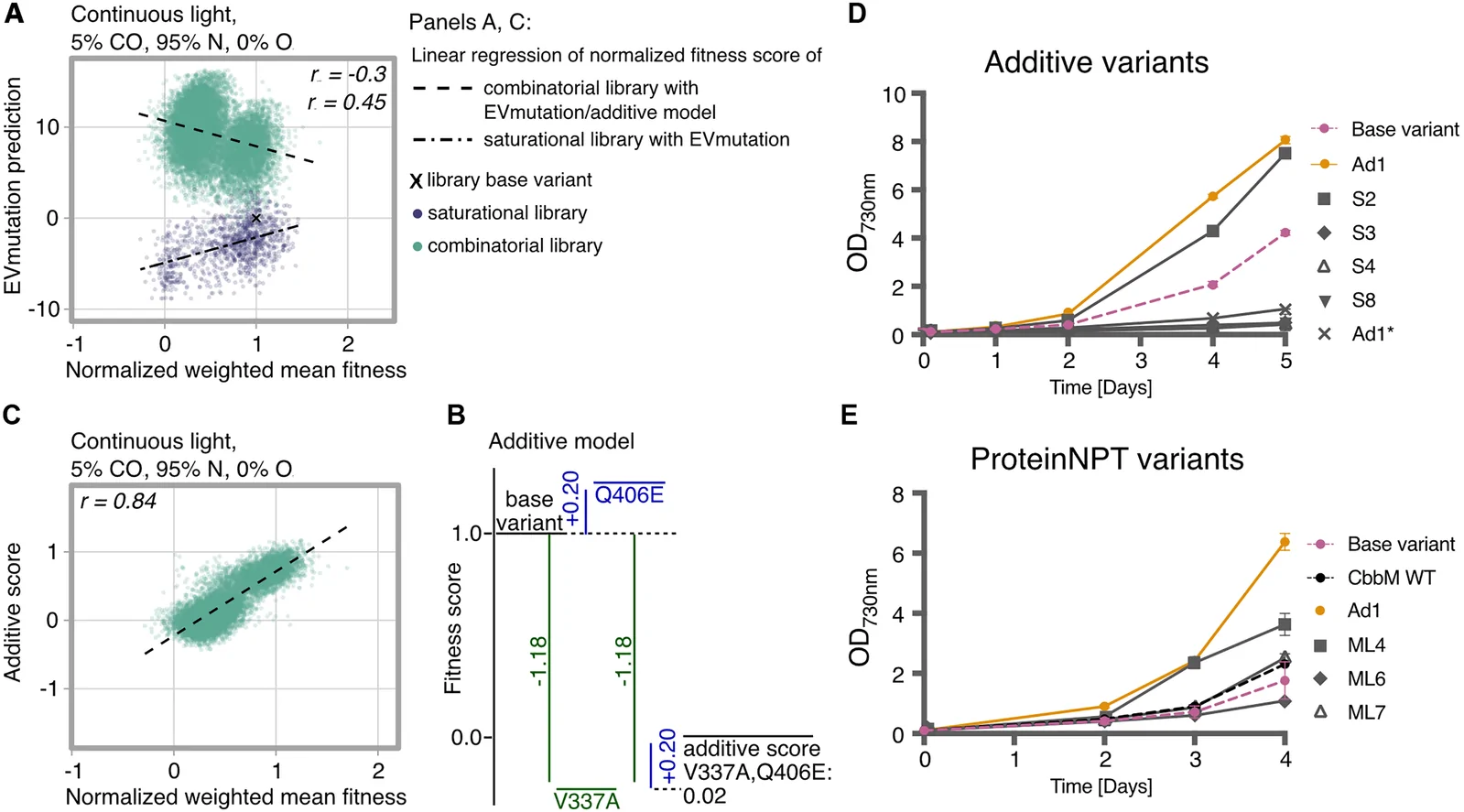
Using labeled fitness data to design CbbM variants. (**A**) Comparison of EVmutation predictions and normalised fitness scores for single-site variants and combinatorial variants. (**B**) Example of additive model for predicting fitness of multi-site variants. (**C**) Comparison of additive model predictions to the normalised fitness scores of combinatorial variants. (**D**) Batch growth of selected second-generation CbbM Synechocystis clones created by the additive method at 5% CO_2_, 75% N_2_, 20% O_2_. CbbM_base: S162P, G422R. Ad1: H127V, K247A, H251A, Q392E, S432T, W114I + S162P, G422R. Ad1* (Ad1 mutations in CbbM_wt background): H127V, K247A, H251A, Q392E, S432T, W114I. S2: V84R, K85N + S162P, G422R. S3: V84D, K219I, D315T + S162P, G422R. S4: F90C, W114K, V256L, M331I + S162P, G422R. S8: V84D, F90C, W114K, Q128D, K219I, V256L, M331I, H344S + S162P, G422R, (**E**) Growth of selected second-generation CbbM Synechocystis clones created by the ProteinNPT method at 5% CO_2_, 75% N_2_, 20% O_2_. CbbM_base: S162P, G422R. Ad1: H127V, K247A, H251A, Q392E, S432T, W114I + S162P, G422R. ML4: H126N, Q142D, K261A, H265A, Q406D, S446I + S162P, G422R. ML6: H126N, Q142D, K261A, H265A, Y333V, Q406D, S446I + S162P, G422R. ML7: W128I, Q142D, K261A, H265A, Q406D, S446I + S162P, G422R. Cultivations shown in (D) and (E) were performed in 6-well plates.

The CbbM fitness data allowed us to test the ability of the zero-shot models EVmutation, DeepSequence and an MSA Transformer ensemble (*59*) to predict fitness scores based on protein sequence. EVmutation is a statistical model capturing site-specific amino acid biases as well as co-dependencies in each pair of sites. DeepSequence and MSA Transformer are neural networks based on a variational autoencoder and a transformer architecture, respectively. Fitness predictions from all three models were highly correlated (fig. S9) and all performed well in predicting the effect of single amino exchanges (r = 0.38–0.45), in line with their reported results on benchmarking datasets (*48*) (Fig. 3A, fig. S10). However, predictions of all models correlated negatively with experimentally determined fitness of higher order variants (Fig. 3A, fig. S10). The inclusion of exchanges V323A and V323S in many combinatorial mutants, which was universally negative despite predictions, has an outsize effect on correlation to combinatorial library data. Removal of variants containing V323 mutations improved fitness predictions [Pearson’s r increased from −0.30 to +0.24 for EV mutation; (fig. S11)].

### Using labeled fitness data to guide rubisco engineering

To assess whether the CbbM fitness dataset could inform a second round of CbbM mutagenesis, we tested both naive and machine-learning guided protein engineering approaches. We first constructed a simple additive model that assumes independence and additivity of fitness effects from single-site exchange variants. This additive model predicted the fitness scores of the library’s higher-order variants well (Fig. 3, B and C, fig. S12, data file S1). Inspired by this model, we tested whether combining positive exchanges would further increase fitness. We created six additive CbbM variants where we combined single-site exchanges: S2 (V84R, K85N), S3 (V84D, K219I, D315T), S4 (F90C, W114K, V256L, M331I), S8 (V84D, F90C, W114K, Q128D, K219I, V256L, M331I, H344S), and two variants where we added positive single-site exchanges to high-fitness combinatorial variants: Ad1 (H127V, K247A, H251A, Q392E, S432T, and W114I) and Ad2 (H127V, K247A, H251A, Q392E, S432T, W114I and K346I). We next used the light-weight model neural network MoCHI (*60*) (fig. S13). Since MoCHI predictions were not more accurate than the additive model, we did not use this predictor to design new variants. Finally, we trained ProteinNPT on the entire experimental fitness dataset. ProteinNPT is a non-parameteric transformer that learns from experimental data connected to CbbM sequences as well as MSA Transformer predictions of the same sequences (*59*, *61*). After training on single-exchange variants, ProteinNPT could predict the fitness of the combinatorial mutants well (fig. S14). From 200 variants predicted by ProteinNPT to have high fitness scores, we selected 11 (ML1-ML11) to test experimentally. These variants contained between 6 and 12 mutations compared to CbbM_base (table S6). We then transformed the CRISPRi*_rbcLSX_* strain with all second-generation CbbM variants and cultivated the strains individually.

Additive variants S2 and Ad1 showed significantly higher growth than CbbM_base and CbbM_wt, and variant Ad1 exceeded growth of all other variants in this study (Fig. 3D). Furthermore, the Ad1 variant exhibited enhanced growth in 1% CO_2_ and ambient air conditions compared to CbbM_base and other first-generation variants (fig. S15). Among ProteinNPT variants, two showed higher growth than CbbM_base, ML4 and ML7, each with six substitutions. However, most *Synechocystis* strains containing second-generation CbbM variants did not grow better than *Synechocystis* containing CbbM_base. Of the additive variants, three of six showed growth similar to a negative control lacking a functional rubisco (S3, S4, S8), and of the ML variants 8 of 11 did not impart growth. Thus, neither the additive model nor the ProteinNPT-assisted model had high accuracy in extrapolating fitness values outside the range present in the dataset. Here again epistasis complicated protein engineering. While variant Ad2 differed from variant Ad1 in only a single exchange which independently was beneficial (K346I), this variant had severely impaired growth. To assess whether adaptive mutations were transferable to other genetic backgrounds, we created a composite mutant incorporating all substitutions from Ad1 but excluding the base variant mutations (S162P and G442R). This variant failed to grow under elevated CO_2_ conditions, indicating that the Ad1 exchanges are specifically beneficial for the CbbM_base starting variant and not CbbM_wt.

### Catalytic parameters of higher-order rubisco variants

**Fig. 4.**
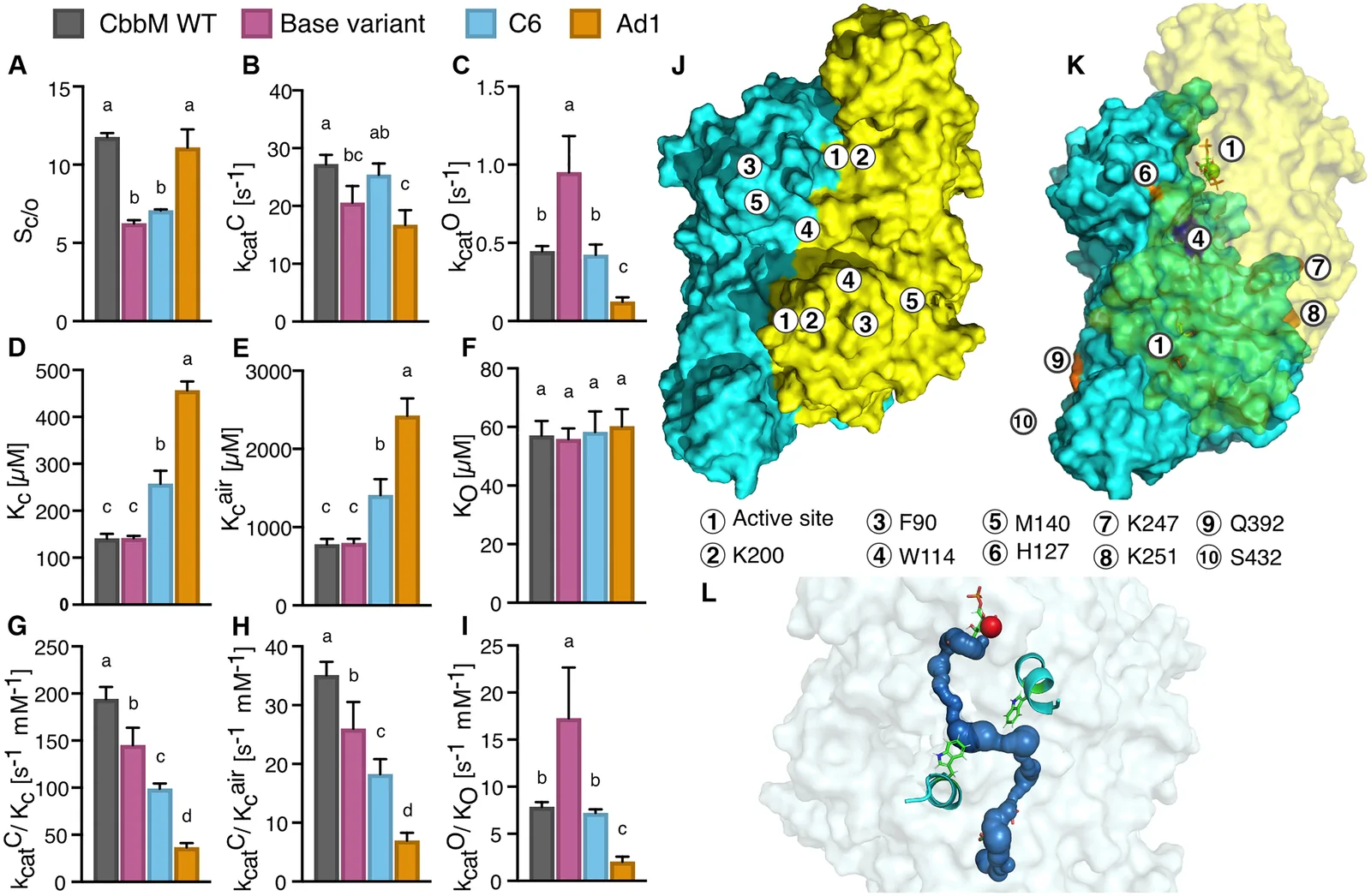
In vitro determined catalytic parameters of select CbbM variants. CbbM_base: S162P, G422R, C6: H127V, M140A, K247A, H251A, Q392E, S432T + S162P, G422R, Ad1: H127V, K247A, H251A, Q392E, S432T, W114I + S162P, G422R. (**A**) Selectivity values of selected CbbM variants. (**B**) Carboxylation rate constant (*k*_cat_^C^). (**C**) Oxygenation rate constant (*k*_cat_^O^). (**D**) Binding constant of CO_2_ in absence of oxygen, (**E**) of CO_2_ in air, (**F**) binding constant of O_2_. (**G**) Catalytic efficiency of carboxylation reaction in absence of air, (**H**) of carboxylation reaction in air, (**I**) catalytic efficiency of oxygenation reaction. All catalytic parameters were determined using a ^14^CO_2_ fixation assay with four replicates. Statistical significance was assessed using a one-way ANOVA followed by Tukey’s multiple comparisons test (**J**) Visualization of important residues and active site in a CbbM model (AlphaFold). The dimers are shown in blue and yellow (**K**) Visualization of CbbM model displaying the location of residues mutated in variant Ad1. (**L**) Residue W114 (green) in CbbM constricts a tunnel (blue) in the dimer interface, connecting the two active sites.

We next determined the catalytic parameters of C6, the highest fitness zero-shot variant, and Ad1, the highest fitness 2nd generation variant, as well as CbbM_base, and CbbM_wt using an in vitro ^14^CO_2_ assay (*62*–*64*). The variants were expressed in *E. coli* and purified without removing the C-terminal polyhistidine tag, which was previously reported to not affect enzyme activity (*22*). Kinetic parameters for CbbM_wt were published previously, and our results were similar (*21*). CbbM_base exhibited a 30% lower *k*_cat_^C^ and 55% higher *k*_cat_^O^ compared to CbbM_wt. Thus, the starting point for the library had impaired kinetics compared to CbbM, which was not apparent in our previous fitness screen, presumably because expression of CbbM variants in that study was from an episomal plasmid with a strong promoter (*40*). Both C6 and Ad1, which imparted better growth in *Synechocystis* cultures than CbbM_base, were distinguished by a significantly lower k_cat_^O^ and k_cat_^O^/K_O_ than CbbM_base (Fig. 4C). Additionally, Ad1 had a significantly increased S_C/O_ (Fig. 4A). Ad1 contains the exchange W114I, which may affect gas tunnels (Fig. 4, J to L). Carboxylation turnover k_cat_^C^ was not as markedly different; C6 had a 25% higher k_cat_^C^ and Ad1 had a 15% lower k_cat_^C^ than CbbM_base. K_c_ was increased in both variants. To test the fitness effect of a higher k_cat_^C^, we grew CbbM_base, CbbM_wt, and Ad1 in batch cultures (5% CO2 95% N2). All exhibited comparable growth (fig. S15B). These results indicate that selection under anaerobic conditions was not driven solely by differences in k_cat_^C^. C6 contains the exchange M140A that is not present in Ad1, and alanine in this position is present in rubiscos from *R. rubrum*, *S. oleracea,* and *Synechocystis* sp. PCC 6803 (fig. S1). A result of these changes in individual parameters is that k_cat_^C^/K_C_ and k_cat_^O^/K_O_ were correlated across the variants (Fig 4, G to I), while k_cat_^C^ and k_cat_^O^ were not correlated.

We also tested thermal stability and oligomerisation of selected CbbM variants. We found that variants displaying increased fitness in aerobic growth generally had a lower melting temperature as measured by NanoDSF (fig. S16). This reduced stability may reflect an altered protein-protein affinity at the dimer interface causing a reduction in protein rigidity. One such example could be the exchange W114I, which may open up cavities in between the dimers (fig. S7, C and D). To see if the mutations had any effect on the quaternary structure (*24*), we performed Mass photometry and compared Ad1 to CbbM_base. Both variants were found to be present as dimers in 95–97% of the population (fig. S17).

### Physiological effects of CbbM variant Ad1 on *Synechocystis*

**Fig. 5.**
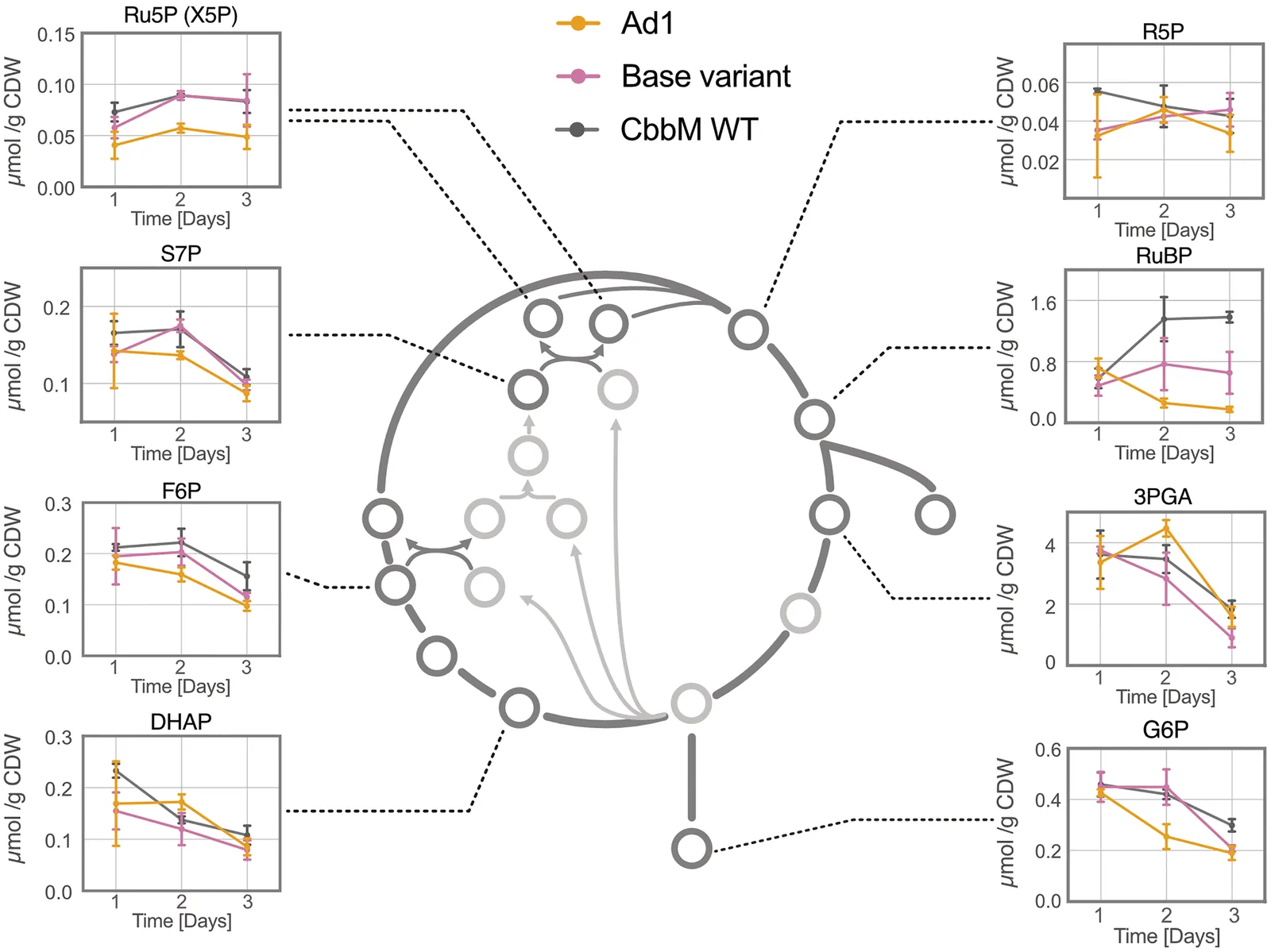
Calvin cycle metabolite levels in CbbM dependent *Synechocystis* strains over three days. Ad1 (yellow), CbbM_base (pink) and CbbM_wt (gray). All CE-MS samples were collected from three biological replicates. Ru5P, ribulose 5-phosphate; X5P, xylulose 5-phosphate; R5P, ribose 5-phosphate; RuBP, ribulose-1,5-bisphosphate; 3PGA, 3-phosphoglycerate; G6P, glucose 6-phosphate; DHAP, dihydroxyacetonephosphate; F6P, fructose 6-phosphate; S7P, sedoheptulose 7-phosphate. The growth condition was 5% CO_2,_ 95% air.

To better understand the improved growth rate of the Ad1 strain we performed capillary electrophoresis mass spectrometry metabolomics on the Ad1, CbbM_wt and CbbM_base strains during three time points in a batch cultivation, and quantified metabolites in the Calvin cycle (Fig. 5, data file S2). We observed that most Calvin cycle intermediates were lower in the Ad1 strain throughout the cultivation. In particular, RuBP was significantly reduced in the Ad1 strain compared to CbbM_WT and CbbM_base. This suggests that there is less of a bottleneck in RuBP conversion for Ad1 compared to CbbM_wt and CbbM_base. Levels of 3-phosphoglycerate (3-PGA) were higher in the Ad1 strain, though the magnitude of change was not as significant as for RuBP. The 3-PGA levels in the strains correlated with their observed growth rates. The concentration of 2-PG was below the detection limit of the CE-MS.

To complement metabolomics analysis, we also collected quantitative mass spectrometry proteomics data of strains CbbM_base, Ad1, and CbbM_wt after repression of native rubisco. The Ad1 strain showed upregulation of multiple ribosomal subunits, in agreement with a faster growth. However, most proteome changes were under the statistical significance threshold, so that there were no obvious mechanisms for the improved growth (fig. S18).

Finally, we used a Clark type electrode to measure photosynthesis and respiration rates of the same strains, comparing it with the CRISPRi_rbcLSX_ strain without a CbbM variant. Net oxygen evolution rates were measured in the dark before illumination, during illumination, and after DCMU addition. In all cases, strains containing CbbM exhibited higher O_2_ uptake than the strain without CbbM, likely due oxygenation activity by CbbM. However, there were no significant differences between the CbbM_base, Ad1, CbbM_wt variants (fig. S19), suggesting that the enhanced growth rate of Ad1 is not due to enhanced respiration.

## DISCUSSION

Here we report fitness data on more than 17,000 mutants of the form II *Gallionella* rubisco. When taken together with other high-throughput, labeled fitness screens (*29*, *65*), this dataset can form the basis for training rubisco models. As an alternative to full-protein DMS and error-prone PCR methods, we employed zero-shot predictors based on a rubisco MSA containing 10,492 sequences (Meff = 2,438) (fig. S20). We reasoned that this approach was more likely to result in rubiscos of higher fitness. It is noteworthy that, even though our zero-shot library covered just a fraction of CbbM residues (67/470, 14%), approximately 8% of zero-shot variants showed an increased fitness compared to CbbM_base. Zero-shot predictors were accurate in predicting fitness effects of single-site mutants, but less accurate at predicting the fitness of multi-site variants. A large factor was the presence of a single epistatic exchange in the multi-site library, though when this was accounted for prediction was still only moderate. Poor prediction of epistasis could indicate a bias in the underlying MSA used by these models, where diversity of sequence is more critical than the MSA depth (*59*). To mitigate this problem, emerging zero-shot predictors for proteins enhance accuracy by incorporating protein structures as well as MSAs (*66*, *67*).

We used our labeled fitness dataset to test two approaches for enhancing fitness of CbbM_base (fig. S20). We compared a directed evolution-like recombination strategy, in which beneficial mutations are combined, and a machine-learning approach, where the partial fitness landscape is learned and used to predict fitness of new variants. Previously, it was estimated that machine-learning guided directed evolution marginally outperforms a recombination approach, in that mutants with optimal fitness could be achieved with fewer rounds and screened variants (*68*). In our study, the fraction of 2nd generation variants that were functional and higher fitness than CbbM_base were similar between the two methods (approximately 50% and 15%, respectively). It is likely that the CbbM fitness could be further enhanced by additional rounds of validation, generation and sampling, using emerging ML approaches (*37*, *69*).

Cell-based fitness screens allow for broad sequence coverage, but cell growth can be affected by multiple enzyme features. In this work, it is likely that fitness of *Synechocystis* is related to the oxygenation rates of CbbM variants. Ad1 and C6, which both imparted faster growth than CbbM_base, had lower oxygenation turnover k_cat_^O^ and oxygenation efficiency k_cat_^O^/K_m_^O^. There was also a clear trend that in vivo fitness gains of mutants was more enhanced in the 20% O_2_ growth condition compared to 0% O_2_ condition, which implicates CbbM oxygenation activity. Though we did not purify and characterize additional enzyme variants, fitness-enhancing mutations were often found at the dimer interface, such as at W114, where CAVER analysis found possible substrate access tunnels for gas entry. Recently, it was shown that minimal mutations to the hexameric RbcL_6_ from *Gallionella GWS1B* could shift the quaternary structure to an L_2_ dimer, which also significantly increased the apparent O_2_ binding constant (*24*). Replacements that affect the flexibility of loop 6 also affected fitness, with stabilizing mutations having a positive effect, as has been reported previously for other rubiscos (*28*, *70*, *71*). A recent directed evolution study of the RbcL_6_ from *Gallionella GWS1B* found that growth selection in an RDE *E. coli* resulted in multiple mutations in the dimer interface that lowered oxygen sensitivity of the enzyme (*22*). As the MSA used to train the zero-shot predictors contains thousands of rubiscos that are likely more resistant to oxygen than CbbM, the fitness predictions may be expected to tend toward oxygen resistance. However, other factors can affect observed fitness, such as higher solubility. While western-blotting and mass-spectrometry proteomics showed similar or even reduced expression of selected CbbM, we cannot rule out that other high-fitness variants had higher solubility, and that even small changes in solubility could affect fitness in a rubisco-dependent strain.

In our previous work, we identified CbbM_base as having similar fitness to CbbM_wt. In this study, where all CbbM variants were expressed from a weaker promoter, there was a small but noticeable reduced cell fitness of CbbM_base compared to CbbM_wt when grown in 5% CO_2_ and air, though no significant difference when grown in 5% CO_2_ and nitrogen (Fig. 2E, fig. S11B). Therefore, it is important for growth-coupled fitness screens that total enzyme activity is not too high as to mask changes in kinetic parameters. The cyanobacteria system complicates selection of mutants based on carboxylation, particularly if this parameter is mechanistically linked to oxygenation efficiency (*8*). Even in a low O_2_, high CO_2_ gas feed, *Synechocystis* growing with CbbM has a proteome consistent with carbon limitation (fig. S2 and table S3), which may be a result of photorespiration occurring from in situ generated O_2_. We were able to isolate mutants with significantly decreased oxygenation turnover (k_cat_^O^) with only marginally reduced carboxylation turnover (k_cat_^C^). However, reduced k_cat_^O^/K_M_^O^ was accompanied by a reduced k_cat_^C^/K_M_^C^ in both cases. Since all growth conditions contained 5% CO_2_, the enzyme may be CO_2_ saturated.

Cell physiology characterization showed Ad1 supplies higher 3-PGA levels in *Synechocystis.* In addition to an immediate benefit for biomass formation, 3-PGA is a known co-activator of RbcR, which activates bicarbonate and CO_2_ transporters in *Synechocystis* (*72*). However, proteome differences between CbbM strains were minor, showing upregulation of ribosome components in the faster growing Ad1 strain, but not increased levels of bicarbonate transporters. The oxygenation activity of CbbM may obscure detection of changes in light-enhanced respiration rates that could contribute to the observed growth. It is also possible that the Ad1 CbbM has a higher degree of activation in vivo than CbbM_wt and CbbM_base, so that there are more active sites per protein mass, a state not captured in in vitro characterisation. A putative rubisco activase is present in the *Gallionella* genome, but was not included in the library or validation strains. Further study is needed to more conclusively link reduced oxygenation to enhanced growth, as well as to characterise more high-fitness CbbM variants identified in library screening.

Our extensive fitness dataset could form the basis for continued engineering of rubisco to obtain both fast and selective variants. Enhanced rubiscos have implications for bioproduction outside of a cyanobacteria platform. In metabolic engineering applications with other CO_2_-fixing bacteria, where product synthesis may be limited by rubisco activity (*5*), fast rubiscos with poor CO_2_ affinity may be good candidates for cultivation at high CO_2_ conditions to exploit high k_cat_ or, in the case of photosynthetic hosts, compartmentalisation in carboxysomes or synthetic organelles. Fast rubiscos optimized for activity in oxygenic photosynthetic cyanobacteria may be more suitable for plants due to their closer evolutionary relationship. Similar to C3 plants, cyanobacteria have high levels of Calvin cycle sugar phosphates, though the relative abundances of some of these are different, in particular SBP and DHAP (*73*). However, transfer of heterologous rubiscos to plants will require integration of the carbon concentrating components, which is a current outstanding challenge. Furthermore, the extent of limitation from rubisco kinetics is highly species specific. Recently, it was shown that the form II rubisco from *R. rubrum,* which has a significantly lower k_cat_ than the rubiscos reported here, was sufficient to sustain potato growth similar to wild type at elevated CO_2_, suggesting enzyme turnover is not limiting (*74*).

## MATERIALS AND METHODS

### Experimental design

The objective of this study was to identify variants of *Gallionella* rubisco, CbbM, which increased the growth rate of a cyanobacterial host strain, *Synechocystis*, when replacing endogenous *Synechocystis* rubisco and compared to a starting CbbM variant. We performed controlled laboratory experiments including the pooled screening of a library of mutant variants at different growth conditions that should select for different properties of the screened enzyme. In a next step, promising variants were characterized further both in vivo as well as in vitro.

### Library design, zero-shot predictions, and ProteinNPT

All scripts used for library construction and analyses are available on GitHub and Zenodo (https://github.com/ute-hoffmann/CbbMLargeLibrary and https://doi.org/10.5281/zenodo.17774822).

The screened library consisted of a saturational library, in which single amino acid exchanges were introduced into each separate variant, and a combinatorial library, into which up to seven amino acid exchanges were introduced at the same time. For the saturational/DMS part of the library, we predicted the fitness of rubisco variants with a single amino acid exchange using the EVmutation (*43*, *44*) web server (https://v2.evcouplings.org/) and DeepSequence (*46*). We used the amino acid sequence of the N-terminally Strep-tagged CbbM_base variant (GenBank identifier OGS68397.1 with amino acid exchanges A230E and G422R) as input for all fitness predictions (EVmutation, DeepSequence, MSA Transformer, ProteinNPT, MoCHI). To predict amino acid exchange effects, we used the EVmutation web server (https://v2.evcouplings.org/) with default settings (bitscores 0.1, 0.3, 0.5, 0.7; 5 search iterations; sequence database UniRef90; position filter 70%; sequence fragment filter 50%; removing similar sequences 90%; downweighting similar sequences 80%; statistical inference model: pseudolikelihood maximization). The web server identified a bitscore of 0.3 as optimal for alignments, which led to a multiple sequence alignment of 10,492 sequences (number of effective sequences Meff = 2,438; number of redundancy-reduced identified homologs divided by confidently aligned positions in the alignment Neff/L = 6.5). This alignment was used for all subsequent tools which relied on a MSA. To run DeepSequence, we adapted code by Hsu *et al*. (*47*) for training the DeepSequence model, used a conda environment provided by Wittmann *et al*. (*36*) and adjusted the DeepSequence code slightly. We picked the subset of residue positions for which the EVmutation epistatic score was above 1.0 or which were among the top 100 predictions of DeepSequence and a EVmutation epistatic score above −1.0. In addition to EVmutation/DeepSequence zero-shot predictions, part of the library consisted of lysine residues in close proximity to residues that promote chemical acetylation (table S1). These were chosen based on lysine modification acetylation as a general regulator of enzyme activity in bacteria (*50*).

Exchanges to be included in the combinatorial library were chosen as described previously on the basis of “in silico evolution” using either EVmutation or DeepSequence as a fitness predictor. For EVmutation, we used 1000 trajectories of in silico evolution with 500 steps for a first step, in which all possible exchanges could be introduced and a second step, in which only a subset of 20 residues, which occurred most frequently during the first step, could be exchanged. We ran DeepSequence predictions on a Google Cloud Product n1-highmem-2 virtual machine with an NVIDIA T4 GPU. When using DeepSequence as fitness predictor, we performed 100 trajectories of in silico evolution for 500 steps with all possible amino acid exchanges. This was followed by another 100 trajectories of in silico evolution for 200 steps after limiting to the same subset of most frequently occurring 20 exchanges as mentioned above. All trajectories were run at a temperature of 0.03. The most frequently occurring amino acid exchanges predicted by the latter rounds of in silico evolution were included in the combinatorial library.

Zero-shot predictions for the entire library were performed using an ensemble of the MSA Transformer model based on five different seeds using code provided by (https://github.com/OATML-Markslab/ProteinNPT/) on an AWS EC2 g6e.xlarge instance with an NVIDIA L40S Tensor Core GPU. When fitness values were available, ProteinNPT was, in a first step, trained on fitness data of the complete saturational part of the library in the multi-objective setting. This training dataset included fitness values for the single amino acid exchange variants underlying the combinatorial library. The trained model was used for predicting combinatorial variants for which normed fitness values were available. In a second step, ProteinNPT was trained on the whole library and the resulting model was used to predict the fitness of variants which seemed to be promising based on the fitness effect of the separate exchanges. These numbers were compared with the naive additive model and based on both, variants to be tested experimentally were picked. For training of ProteinNPT, the same AWS EC2 instance as mentioned above was used. Additionally, fitness values of the single amino acid exchange variants were used to train MoCHI (*60*). MoCHI was trained separately on the data sets corresponding to the three tested growth conditions. For transformations, the “SumOfSigmoids” option was used. These three separate models were used to predict fitness scores for the combinatorial variants of the library. MoCHI was run on a desktop PC without GPU.

### Library synthesis and plasmid preparation

Libraries were synthesized by GenScript (Amsterdam, Netherlands). CbbM variant libraries were inserted into a plasmid designed for genomic integration of the expression plasmid at the *slr0168* locus of *Synechocystis*. The plasmid map of the respective “base” plasmid is uploaded on GitHub (https://github.com/ute-hoffmann/CbbMLargeLibrary). After receiving the plasmid pools, libraries were transformed via electroporation into CopyCutter EPI400 *E. coli* cells (Lucigen, epicentre) and colonies corresponding to a 70X - 100X coverage of each library were pooled in LB medium separately for the combinatorial and the saturational library. Aliquots were frozen in 25% (v/v) glycerol, 0.5X LB and aliquots were kept at −80°C until further usage. For plasmid preparation, one aliquot per library was thawed in 50 ml LB medium containing kanamycin (50 μg/ml) and grown overnight at 37°C. The next day, cultures were diluted 1:100 in LB containing 1X CopyCutter solution (Lucigen, epicentre). After 4 hours of growth in baffled Erlenmeyer flasks (37°C), cells were harvested and plasmid prepared using MiniPrep kits (Thermo Scientific).

### PacBio sequencing

To assign the N20 barcodes to mutant variants, plasmid DNA extracted from the *E. coli* mutant library was sequenced. To do so, induction of a high plasmid copy number of the pool of CopyCutter EPI400 *E. coli* cells was performed according to the manufacturer’s protocol. Plasmid was extracted using a GeneJET Plasmid Miniprep Kit (Thermo Scientific) and DNA concentration was measured using a Qubit 1X dsDNA High Sensitivity (HS) assay kit (Invitrogen). Saturational and combinatorial plasmid pools were linearized in separate reactions of three to five μg plasmid DNA with single-cutter FastDigest restriction enzymes EcoRI, SmaI or Alw44I (Thermo Scientific). Linearized plasmid was extracted from agarose gels using the Zymoclean Gel DNA Recovery Kit (Zymo Research). Libraries were pooled according to their theoretical number of members and the pool was purified using AMPure XP beads (Beckman Coulter) with a protocol adapted for large DNA fragments. One volume of AMPure XP beads were mixed with the sample and incubated at room temperature for 10 minutes. Beads were separated on a magnetic rack for 2 minutes and supernatant withdrawn. Beads were washed twice with freshly prepared 80% (v/v) ethanol and incubation periods of 1 minute. After a short spin, remaining ethanol was withdrawn and beads were air dried for 2 minutes. DNA was eluted by adding 0.5 X H2O, incubating it for 10 min with the beads, removing beads from the eluate by keeping the tube on the magnetic rack for 2 minutes and then removing the supernatant. This eluate was handed to the national genomics infrastructure (NGI) Sweden for further library preparation. Adapters were added using the SMRTbell prepkit 3.0. The library was sequenced using a Sequel Sequencing Kit 2.0 and a Sequel SMRT Cell 8 M v3 Tray on a Sequel II machine.

### Bioinformatic analyses of PacBio data

PacBio sequences were mapped using minimap2 v2.28 (r1209) (*75*) and its CCS-specific preset to a 1,675 bp long template spanning the promoter region (P*_trc_*), the coding sequence of *cbbM* and a small region up- and downstream of the N20 barcode. The resulting sam-file was filtered for aligned reads with a mapping quality above 5 (MAPQ >5) using the samtools v1.13 (*76*) view command. Further processing was mainly performed using a Jupyter notebook for Python 3.9.7 (PacBio barcode processing.ipynb). First, aligned reads were extracted from the resulting sam file and converted into fasta format. To extract coding sequences and barcodes from this file, cutadapt v3.5 (*77*) was run twice using two different sets of paired adapters corresponding to the sequences exactly up- and downstream of the *cbbM* coding sequence and the N20 barcode, respectively. Untrimmed reads and barcode reads shorter than 15 nt were discarded. Coding sequences were assigned to barcodes according to the PacBio read identifier and coding sequences were translated to protein sequences. Protein products shorter than 300 amino acids were discarded. For barcodes occurring more than once, the assigned coding sequences were compared and the sequence occurring most frequently was used as consensus sequence. In case there were ties, these barcodes were discarded. All remaining barcodes were combined into one file, amino acid exchanges in comparison to the CbbM sequence that was underlying the library (CbbM with A230E and G422R) were determined and the combination of barcodes and assigned mutations were formatted to be compatible with downstream applications. In total, 235,598 barcodes were identified. Of these, 205,150 barcodes were assigned to 17,839 mutant variants expected based on the initial library design. We expected 17,983 mutant variants from the initial library design – 1,805 variants belonging to the saturational part, 16,200 to the combinatorial, which are partially represented in the saturational part. The remaining barcodes represented mutant variants longer than 300 amino acids covered by more than one barcode. All variants expected on base of the library design and missing in the *E. coli* library belonged to the combinatorial library.

Illumina sequencing data from the library cultivation at continuous light and a gas feed of 5% CO2, 95% N2, 0% O2 were mapped to the resulting barcode library. For further analyses, barcodes were only kept if more than 30 reads from these 30 5% CO2, N2 samples mapped to their sequence. The resulting barcode library consisted of 32,549 barcodes, which represented 14,329 expected mutant variants. Only the 30 barcodes with the highest counts assigned to the library base variant, CbbM with A230E and G422R, were kept in this library. In total, 8,968 mutant variants were represented by more than one barcode.

### Bacterial strains and general culture conditions

A nonmotile glucose-tolerant wild type of *Synechocystis* was kindly provided by P. Kallio, University of Turku. BG-11 medium (*78*) with HEPES buffer (20 mM, pH 7.8) was used for culturing. All cultivations with an anaerobic feed gas 5% CO_2_, 95% N_2_, 0% O_2_ were done in Multi-Cultivator. If not cultivated in a Multi-Cultivator or indicated otherwise, cultures were grown in continuously shaking (160 rpm) Erlenmeyer flasks or 6-well plates at 30°C under continuous white-light (45 μmol photons m^−2^ s^−1^) in ambient enriched with 1% (v/v) CO_2_. Plate cultures were grown on 1% (w/v) bacto-agar BG-11 plates with sodium thiosulfate [0.3% (w/v)]. Anhydrotetracycline (aTc) and antibiotics (kanamycin 50 μg mL^−1^, spectinomycin 30 μg mL^−1^) were added when needed.

### Strain construction *Synechocystis*

The plasmid pool harbouring all library variants was transferred into *Synechocystis* strain CRISPRi_rbcLSX_ by natural transformation. After homologous recombination, this yields strains with the following genomic arrangement: Δ*psbA1* P_J23101_::*tetR* P_L22_::*SPdcas9* P_L22_::*sgRNA_rbcL_1* P_L22_::*sgRNA_rbcL_2* Δ*slr0168* P_trc_::*(N-Strep)*-*cbbM.* To transform *Synechocystis*, 10 μg plasmid DNA was mixed with culture at OD_730_ ∼ 0.8, and incubated for 5 h at 30°C, 30 μE. The transformations were plated on cellulose filters on 1% agar plates containing kanamycin (25 μg/ml) and spectinomycin (25 μg/ml), and incubated at 30°C, 30 μE, and 1% CO_2_ for 10 days. The transformation yielded 60,000 colonies. The combinatorial and saturation parts of the library were combined in a ratio of 10:1. The combined library was mixed in 25% (v/v) glycerol, 0.5x BG-11 (HEPES), snap-frozen, and stored at −80°C until further usage.

All variants to be validated and other second-generation variants were ordered as a gene block and introduced into the pEERM-based library plasmid in XL1 blue *E. coli*. The variants were transformed into the CRISPRi strain via natural transformation and individual colonies were picked and confirmed via colony PCR.

### Pooled growth of *Synechocystis* library

Library cultivations was performed in a 8-tube Multi-Cultivator MC-1000-OD bioreactors (Photon System Instruments, Drasov, CZ) using the pycultivator-legacy package (https://gitlab.com/mmp-uva/pycultivator-legacy) (*79*) and as described (*80*) with the following modifications: light intensity was kept at 300 μmol photons m^−2^ s^−1^. The turbidity threshold was set to OD_720_ = 0.2. The library was cultivated in 8 growth channels, giving 8 replicates per condition, inoculated from the same pre-culture. The pre-culture was inoculated from a full cryostock tube containing the combined combinatorial and saturation library. To minimize bias before CRISPRi induction, the preculture was cultivated for only three days. Two gas conditions at constant light were tested, anaerobic feed gas 5% CO_2_, 95% N_2_, 0% O_2,_ and aerobic 5% CO_2_, 70% N_2_, 20% O_2_. Furthermore, light-dark cycles in the oxygen-containing gas feed were used. After the pooled library had acclimated and reached stable cultivation with regular back-dilutions, the native rubisco was suppressed by addition of aTc (2 μg ml^-1^) to cultivation tubes and feeding bottles, which marked generation 0. Growth data was analyzed using the ShinyMC web application (https://github.com/m-jahn/ShinyMC, v0.1.1) growth rates and doubling time was calculated as mentioned in Hoffmann et al. (*40*). Cells were harvested after approximately the 4th, 8th, and 10th generation by centrifuging 12 ml culture (4,500 x g, 4°C, 10 min). Supernatant was removed completely and cell pellets were frozen at −20°C until further usage for NGS preparation. For continuous light, oxygen-free gas feed, cultivation in one of the channels stopped after the fourth generation due to technical difficulties.

### Library preparation and next-generation sequencing

The NGS library preparation was done as described in Hoffmann *et al*. (*40*) with the following changes: DNA was extracted using a GeneJET Genomic DNA Purification Kit (Thermo scientific) following the protocol for Gram positive bacteria and eluted in 40 μl deionised water. After preparation, the library was run on a NextSeq 2000 system (Illumina) using NextSeq 1000/2000 P2 reagents v3 (Illumina) according to the manufacturer’s instructions.

### Illumina NGS bioinformatic analysis and exploratory data analysis

Sequencing data was analyzed using the Nextflow nf-core-crispriscreen pipeline Miao and Jahn *et al*. (*80*) (commit d9e9b1812b2e093a49f290cd0b04fc407ff1d5a2 from 31st Oct 2024) with the following input deviating from the defaults: the sequence GTCTAGAatcgccgaaagtaattcaactccattaa...TCTAGATGCTTACTAGTTACCGCGGCCA was used for the parameter --five_prime_adapter, the error rate was set to 0.2, filter_mapq was set to 1 and both run_mageck as well as gene_fitness were set to false. Briefly, the pipeline performs read trimming with cutadapt (*77*), maps reads with bowtie2 (*81*), data normalization with DESeq2 (*82*) and calculation of fitness scores for individual barcodes. The fitness of a specific barcode at a given condition was determined as the area under the curve given by the log2FC over time *t* normalized by total cultivation time given in generations as given by (*80*)

$$F=\frac{\mathrm{AUC}(t,{\text{log}}_{2}\mathrm{FC})\cdot 2}{\text{max}\left(t\right)}$$

Hence, the fitness of a specific barcode, *F*, is based on several data points, which is an important first step to reduce sampling bias. Custom R Markdown and python scripts were used for subsequent analyses. Fitness values of barcodes associated with a specific mutant variant were compiled to a weighted mean fitness value *F_wmean_* per mutant variant and condition by weighting according to the Pearson correlation of each specific barcode, *r_i_*, with the rest of the barcodes associated with this variant, and the inverse of the maximum standard error on the log2FC values given for a specific barcode by DESeq2

$$F_{\text{wmean}}=\frac{\sum_{i=1}^{n}\left(F_{i}\cdot w_{i}\right)}{n};w_{i}=\frac{r_{i}}{{\text{lfcSE}}_{i}}$$

This step ensures that results for barcodes which do not correlate well with other barcodes for the same variant, e.g. due to their low abundance and therefore high statistical noise or due to secondary mutations in the respective strain, will be downweighted compared to the remaining barcodes. Adjusted *P* values were calculated by comparing the fitness values of the set of barcodes associated with a specific protein variant and the barcodes associated with the protein variant underlying the complete library (CbbM with S162P, G422R) using the two-sided Wilcoxon signed rank test and Benjamini-Hochberg correction (significance level 0.1).

For plotting, weighted mean log2FC were compiled from separate log2FC values of different barcodes by following the same strategy as described above, i.e. by weighting by the weighting factor *w_i_* given by the Pearson correlation of a given barcode and its maximum standard error on the log2FC.

We adapted the methodology of Miton and Tokuriki (*83*) to analyze the effect of exchanges on intermediate variants. They compared the fitness effect of an amino acid exchange *i* on the starting enzyme background, which would be in our case CbbM_base, and on higher-order, intermediate variants. To do so, they calculated the ratios of the fitness of the variants containing the exchange compared to the base variant fitness $\left(∆F_{\text{base},i}=\frac{F_{\text{base}+i}}{F_{\text{base}}}\right)$ and a higher-order, intermediate variant *j* $\left(∆F_{j,i}=\frac{F_{j+i}}{F_{j}}\right)$. We adjusted their approach as follows: Fitness values below 0.1 were set to 0.1 as we considered them as catalytically inactive, based on the distribution of normed fitness values of K200 variants. Deviating from Miton and Tokuriki (*83*), we considered an exchange with 0.8 < $∆F_{j,i}$ < 1 0.25 as neutral, one with $∆F_{j,i}$ < 0.8 as detrimental and with $∆F_{j,i}$ > 1.25 as beneficial. We adapted these cut-offs on the basis of the value distribution in our dataset.

### Further data analyses

Multiple sequence alignments were created using Clustal Omega (*84*, *85*) and visualized with JalView v2.11.1.4 (*86*). Protein structures were visualized using UCSF ChimeraX v1.8 (*87*) and PyMol v2.5.0 (*88*). The solvent-accessible surface area was calculated using the ChimeraX command “measure sasa” and relative SASA using theoretical MaxASA values by Tien *et al*. (*89*).

### Proteomics mass spectrometry sample preparation and analysis

Proteomics was performed both on the cultivated library pool in the turbidostat and on isolated variants from the validation phase. For the library pool cultivations, approximately half the samples taken for NGS were used. Cell pellets were collected as described for photobioreactor cultivations for NGS sampling (see “Pooled growth of *Synechocystis* library”). They were lysed by bead beating (FastPrep-24 5G lysis machine, MP Biomedicals). Proteomics analysis was performed on a Q-exactive HF Hybrid Quadrupole-Orbitrap Mass Spectrometer coupled with an UltiMate 3000 RSLCnano System with an EASY-Spray ion source. A more detailed protocol can be found described in Hoffmann *et al*. (*40*).

### Western blot, nano differential scanning fluorimetry (nanoDSF) analysis, and mass photometry

Solubility and stability comparison between mutant variants were performed as described in Hoffmann *et al*. (*40*).

For mass photometry, purified protein was disputed to 1–2 μM in 18 mM MgCl2 and 18 mM EPPS buffer and analyzed on a TwoMP mass photometer (Refeyn). Sample well cassettes were set up on a microscopic cover slip mounted on the mass photometer using immersion oil. To focus the instrument with droplet dilution, the above mentioned buffer was used. Oligomeric state populations were recorded using AcquireMP with a 1-minute acquisition time and analyzed with DiscoverMP by quantifying protein binding events and Gaussian fitting. Molecular mass was determined by comparing histogram contrast values to the calibration standard measured on the same day, using bovine serum albumin and thyroglobulin.

### In vitro kinetic characterization of CbbM variants

#### Gene cloning and E. coli production

In preparation for in vitro verification the gene for a select number of variants were introduced into pET28a-6xHis-KmR and transformed into *E. coli* BL21(DE3), described in Hoffmann *et al*. (*40*). For each CbbM variant, a single colony was used to inoculate 20 mL of LB (50 μg/ml Km), and incubated overnight at 37°C. The following day, 200 mL 2YT (50 μg/ml Km) culture were inoculated to an OD of OD600 nm of 0.1. 0.5 mM isopropyl-β-d-thiogalactopyranosid (IPTG) was added to induce protein expression when an OD600 nm of 0.6–0.8 was reached, cultures were moved to 16°C and incubated for 16 h before harvested by centrifugation (3005 g, 4°C, 20 min). The harvested cells were lysed using B-PER and purified using Ni-NTA agarose beads (Qiagen) as described in Hoffmann *et al.* (*40*) The proteins were put in a buffer with 18 mM MgCl2 and 18 mM EPPS, and then bicarbonate was added to 50 mM. The resulting purified proteins were immediately frozen in liquid nitrogen and stored at −80°C until measurement.

#### Enzyme assays

Cell extracts containing CbbM were pre-activated by addition of carrier-free NaH14CO3 to a specific radioactivity to 3.7 x 1010 Bq mol-1 and incubation at 25°C for 20 min. 14CO2-fixation was measured at 25°C in 7 ml septum-capped glass scintillation vials containing 400 μl of Assay Buffer (100 mM Bicine pH 8.1, 20 mM MgCl_2_) and ∼100 W-A units of carbonic anhydrase (C3934 Merck, USA). The reaction mixture was first bubbled with either 100% N_2_ gas or CO_2_- free synthetic air (21% O_2_, 79% N_2_) for 1 h. Next, ^14^CO_2_ ranging from 16 to 500 μM and 1.6 mM of in-house synthesized RuBP (*63*) were added to the vials. Finally, assays were started by the injection of 20 μl of pre-activated cell extract and quenched after 1 min by addition of 0.2 ml of 10 M formic acid. After removal of acid-labile ^14^C by evaporation, acid-stable ^14^C was determined by liquid scintillation counting (Tri-Carb, Revvity USA).

The Michaelis–Menten constant for CO_2_ (*K*_c_) and the maximum carboxylase activity (*V*_max_^c^) was calculated as in Capó *et al*. (*62*). The Michaelis–Menten constant for O_2_ (*K*_o_) were calculated in each biological replicate by a linear fit of the *K*_c_ measurements obtained under 0% O_2_ and under 21% O_2._ The carboxylation turnover rate (*k*_cat_^c^) was calculated by dividing *V*_max_^c^ by the concentration of rubisco active sites, which was quantified from the same protein extracts by 2′-carboxyarabinitol-1,5-bisphosphate (^14^C-CABP, 130 μM final concentration) binding Kubien *et al*. (*64*). Concentrations of CO2 in solution were calculated assuming an acid dissociation constant for carbonic acid (pKa_1_) of 6.11 and a solubility constant for CO_2_ of 0.034 mol l^−1^ atm^−1^ at 25°C and using accurate measures of the pH (NBS scale).

To calculate *S*_c/o_, CO_2_-free protein extracts were obtained as described above, using a Desalting Buffer without NaHCO_3_ and bubbled with 100% N_2_ gas._._ *S*_c/o_ was analyzed by using [1-^3^H] RuBP, following the method of Kane *et al*. (*63*) with the modifications of Capó *et al*. (*62*). The assay was equilibrated with a gas mixture of 99.95% O_2_ and 0.05% CO_2_, and *S*_c/o_ was calculated using a CO_2_/O_2_ solubility ratio of 0.038 at 25°C.

### Protein model prediction and tunnel analysis

The structure of CbbM_wt without N-Strep tag was predicted by homology modelling using YASARA Structure (v.22.5.22.W.64), which identified suitable structural templates through BLAST and PSI-BLAST searches against the Protein Data Bank. Templates were selected based on sequence identity, alignment quality, and structural resolution. Each model was refined using YASARA’s integrated optimization and energy minimization pipeline with the AMBER03 force field in explicit solvent. The resulting models were ranked by YASARA’s Z-score, and the top-scoring model was chosen.

The structure of the designed variants used for tunnel analysis were predicted using the AlphaFold Protein Structure Database prediction server (DeepMind/EMBL-EBI) (*90*) using the CbbM_base protein sequence without N-Strep tag and allowing incorporation of Mg^2+^ ions. The “Multimer” option was selected to enable prediction of the dimeric assembly. Two identical copies of the sequence were provided as input to generate a homodimer model. The server generated multiple sequence alignments using its standard suite of sequence databases and executed the AlphaFold-Multimer prediction pipeline. For each submission, five structural models were produced. Confidence was evaluated using: pLDDT for per-residue accuracy; ipTM and pTM scores for global and inter-chain prediction confidence; Predicted Aligned Error (PAE) maps to assess chain–chain orientation reliability. The highest-ranked model according to AlphaFold’s internal ranking metric was selected for downstream analysis.

The predicted structures were then analysed using Caver web 2.0 (*91*), using residue K200 as a starting point and with the following parameter inputs: probe_radius 0.9, shell_depth 4.0, shell_radius 3.0, clustering_threshold 3.5, max_distance 3.0, desired_radius 5.0.

### Metabolome analysis of CbbM-dependent *Synechocystis* using CE-MS

CRISPRi was induced with aTc (2 μg ml-1) when cultures were at density OD_730_ 0.2. The strains were backdiluted and fresh aTc added after two days. When OD_730_ reached 2 an equivalent of 5 mg DCW was harvested by filtration with 1-μm pore size PTFE disks (Omnipore; Millipore, Billerica, MA,) every 24 h for three days. The filter was washed with ice-cold 20 mM ammonium carbonate. The intracellular metabolites were extracted as previously reported (*92*). Before analysis all solubilized proteins were removed by filtration through a 3 kDa cutoff membrane (Millipore). The metabolites were analyzed using a capillary electrophoresis-mass spectrometry (CE-MS) system (CE, Agilent G7100; MS, Agilent G6230 LC/MSD TOF; Agilent Technologies, Palo Alto, CA, USA). The metabolites were subsequently quantified using standards with known concentrations of each metabolite.

### Oxygen evolution and respiration measurements

Cells were grown to OD_730nm_ 0.5 and harvested at 4000 g for 10 min. The pellet was resuspended in 90% methanol, vortexed and incubated in dark for 20 min. The extraction was centrifuged for 10 min at 10,000 g. The amount of chlorophyll was quantified by absorption at A665nm. A culture with density equivalent to 20 μg/ml Chl was harvested by centrifugation at 3000 g and resuspended in 2 ml media. Oxygen evolution was measured using a Clarktype electrode (Hansatech Instruments) four days after induction by aTc. Before measurements 10 mM of sodium bicarbonate (NaHCO_3_) was added to the cells. Initially, the cells were incubated in dark for 5 min. Next, a saturating light of 100 μmol photons m^−2^ s^−1^ was applied. To estimate the respiration of oxygen in the light, 20 mM DCMU was added (after 10 min, from start). The consumption of oxygen was then measured for 5 min at saturating light. The oxygen evolution rate and respiration rates were then normalized to the chlorophyll content of each sample.
