# Supplementary material for: The fitness landscape of a form II rubisco in a photosynthetic bacterium guides engineering of oxygen tolerance
Source: Sci Adv. 2026 Sep 4;12(36):eaee9222. doi: 10.1126/sciadv.aee9222 (PMC13544237; doi:10.1126/sciadv.aee9222)
Supplement: Supplementary file 1 [file sciadv.aee9222_sm.pdf]

Science Advances
AAAS

Supplementary Materials for

The fitness landscape of a form II rubisco in a photosynthetic bacterium guides engineering of oxygen tolerance

Ute A. Hoffmann *et al.*

Corresponding author: Elton P. Hudson, paul.hudson@scilifelab.se

Sci. Adv. **12**, eaee9222 (2026)
DOI: 10.1126/sciadv.aee9222

The PDF file includes:

Figs. S1 to S20
Tables S1 to S6
Legends for data files S1 and S2

Other Supplementary Material for this manuscript includes the following:

Data files S1 and S2

Supp. Fig 1. (related to Fig. 1) Alignment of parts of the sequence of *Gallionella* sp. (*Gallionella*) Form II rubisco with homologous parts of Form I rubiscos of *Spinacia oleracea* (*Spinacia*), *Synechocystis* sp. PCC 6803 (*Synechocystis*), *Synechococcus* sp. PCC 6301 (*Synechococcus*), *Thermosynechococcus kodakarensis* ATCC BAA-918 (*Thermosynechococcus*), *Chlamydomonas reinhardtii* (*Chlamydomonas*), *Cupriavidus necator* (*C. necator*), Form II rubiscos of *Rhodospirillum rubrum* (*R. rubrum*), *Rhodopseudomonas palustris* ATCC BAA-98 (*R. palustris*), *Gallionella* GWSB1 (primary accession A0A0X1KHE5) as well as Form III rubiscos of *Archaeoglobus fulgidus* ATCC 49558 (*A. fulgidus*) and *Methanococcoides burtonii* DSM 6242 (*M. burtonii*). Shades of blue indicate degrees of similarity. *Gallionella* residue positions included in the large library are indicated by arrows. Numbers above the alignment give the absolute position in the complete alignment.

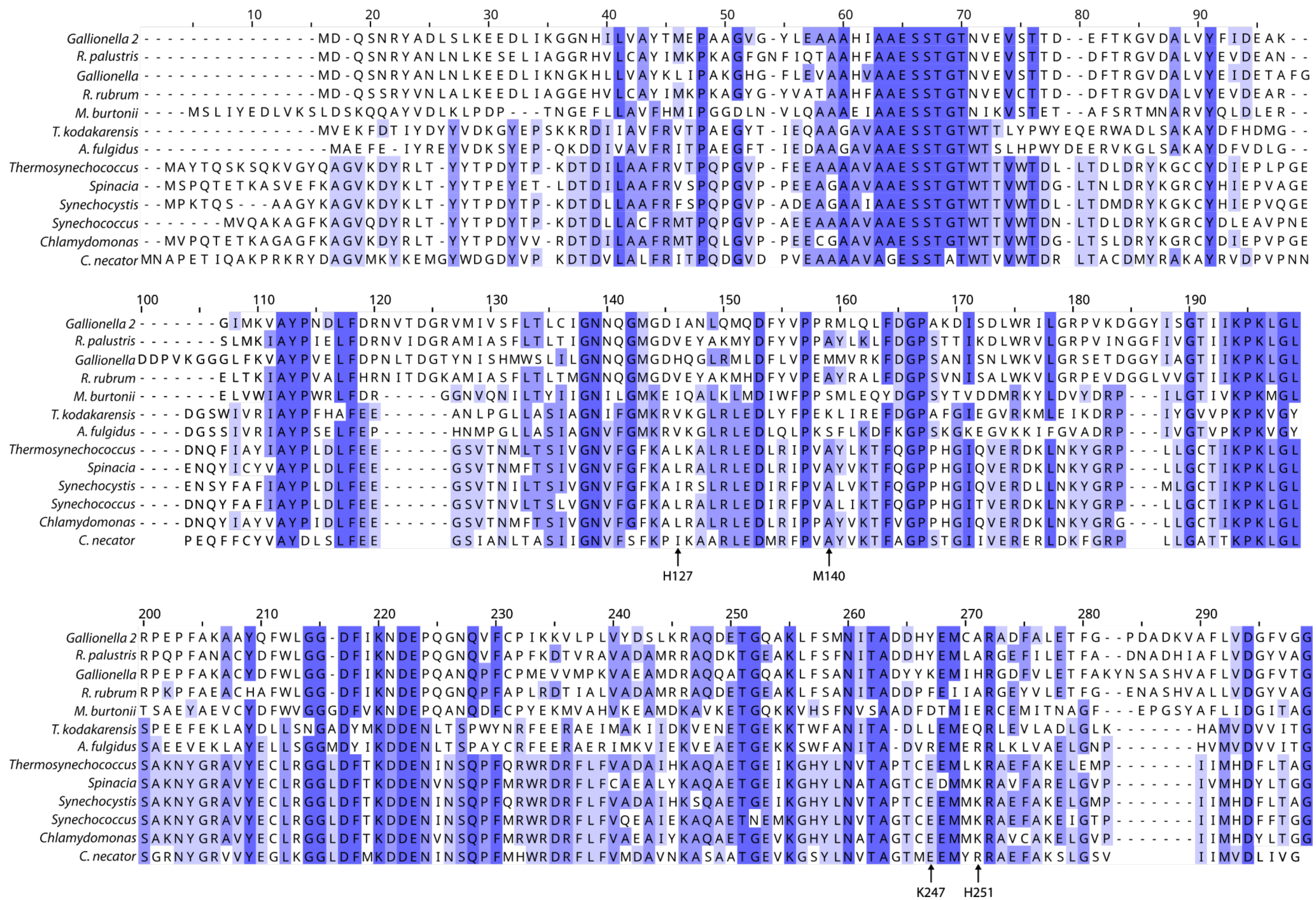

Gallionella 2
R. palustris
Gallionella
R. rubrum
M. burtonii
T. kodakarensis
A. fulgidus
Thermosynechococcus
Spinacia
Synechocystis
Synechococcus
Chlamydomonas
C. necator
H127
M140
K247
H251

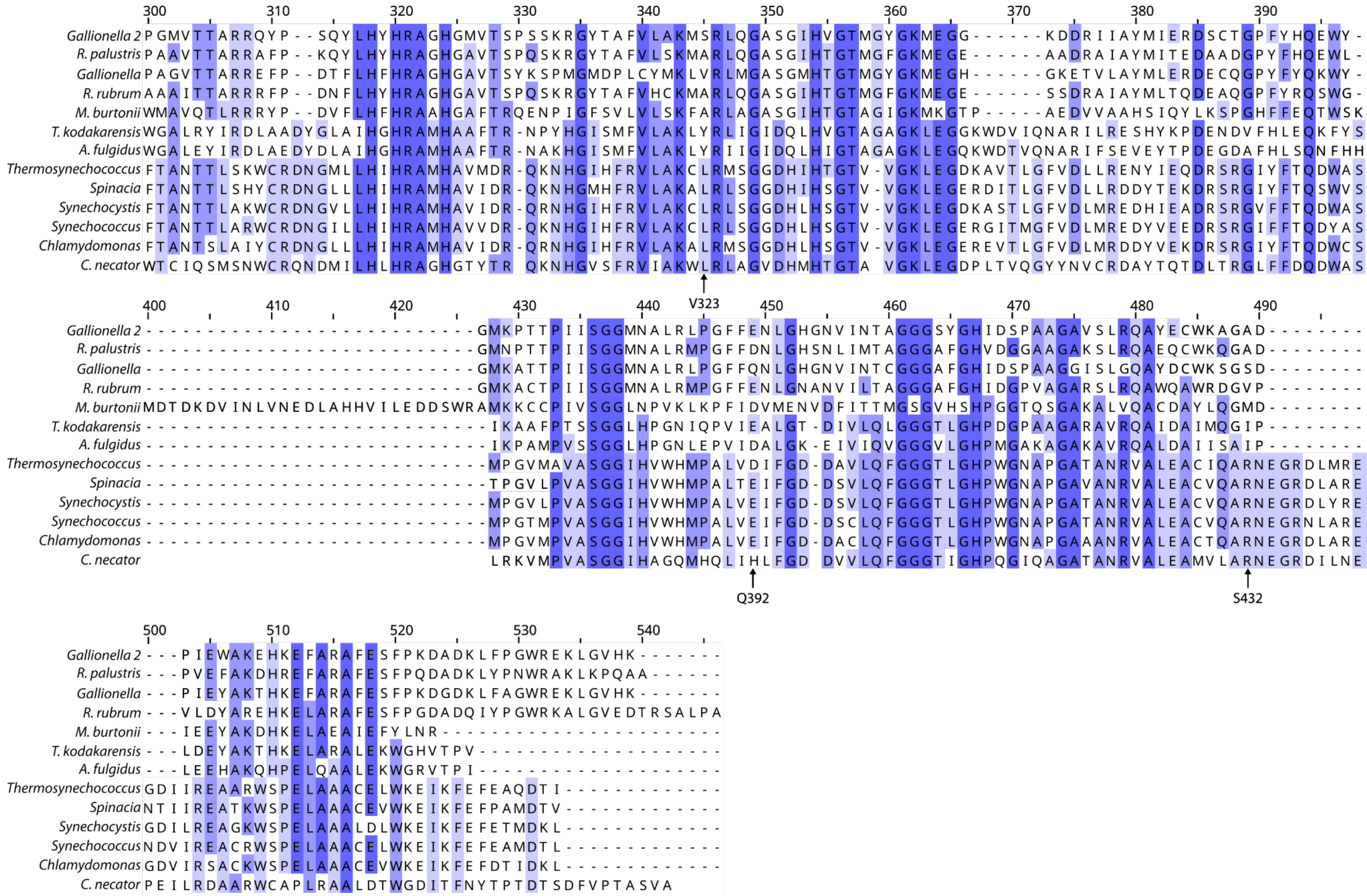
300
310
320
330
340
350
360
370
380
390
Gallionella 2
R. palustris
Gallionella
R. rubrum
M. burtonii
T. kodakarensis
A. fulgidus
Thermosynechococcus
Spinacia
Synechocystis
Synechococcus
Chlamydomonas
C. necator
V323
400
410
420
430
440
450
460
470
480
490
Q392
S432
500
510
520
530
540

Supp. Fig. 2. Plot of all weighted mean log2FC values of all CbbM variants over time, normalized to time point generation 0. The base variant and inactive mutant K214R (K200R when strep-tag is excluded) are highlighted, all other variants shown in light gray.

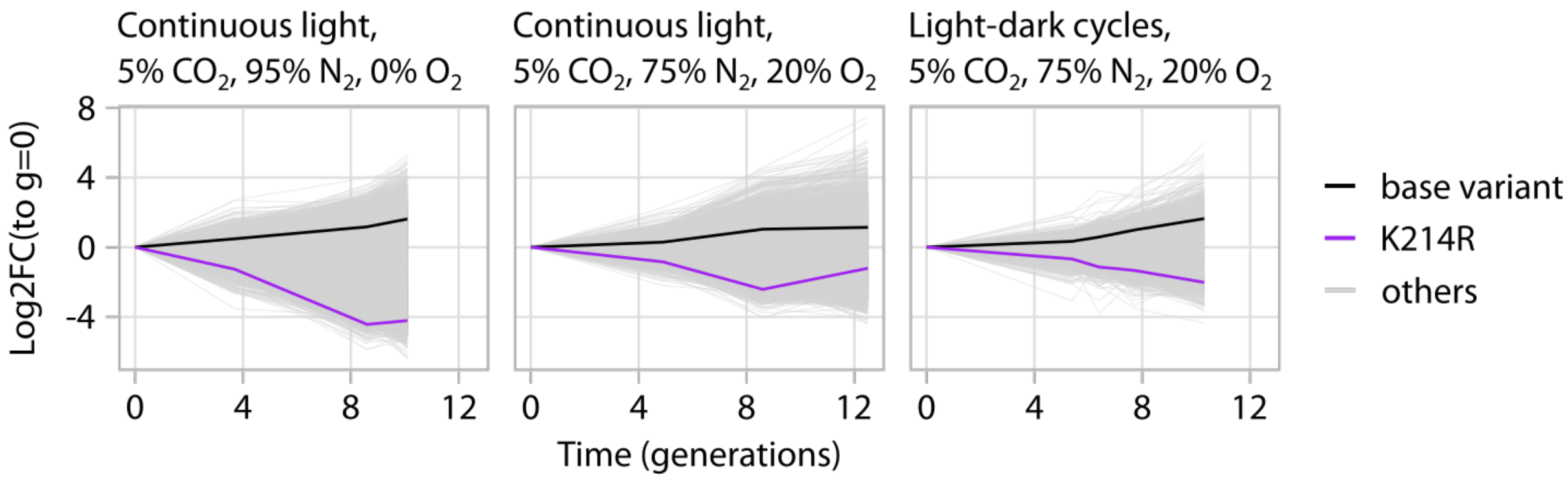

Supp. Fig. 3. Volcano plots of proteomics data comparing samples taken at generation 0 (before induction with aTc) and (**A**) generation 3.7 and (**B**) generation 8.6 in continuous light and a gas feed of 5% CO2, 95% N2, 0% O2. Proteins are labeled with their gene name in case they belong to operons which are known to be of relevance for the carbon-concentrating mechanisms (compare Supp. Table 3). In addition, *dCas9*, *rbcS* and *rbcL* as well *cbbM* are labeled.

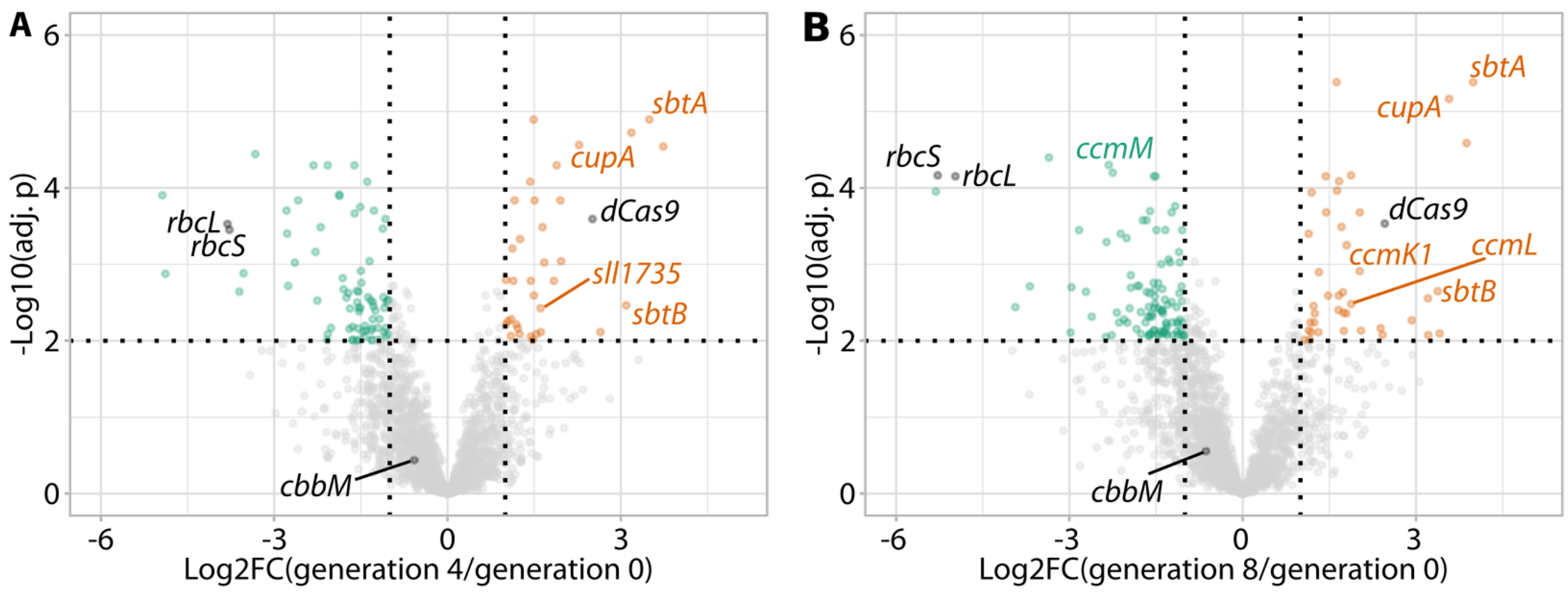

Supp. Fig. 4. Diagnostic plots for pooled growth (related to Fig. 1) (**A**) Histogram showing the frequency of the variance of measured fitness values for each investigated protein variant. (**B**) Histogram showing the frequency of adjusted *p* values for different investigated variants. (**C**) Scatter plots showing the relationship of the number of barcodes per investigated variant to adjusted *p* values.

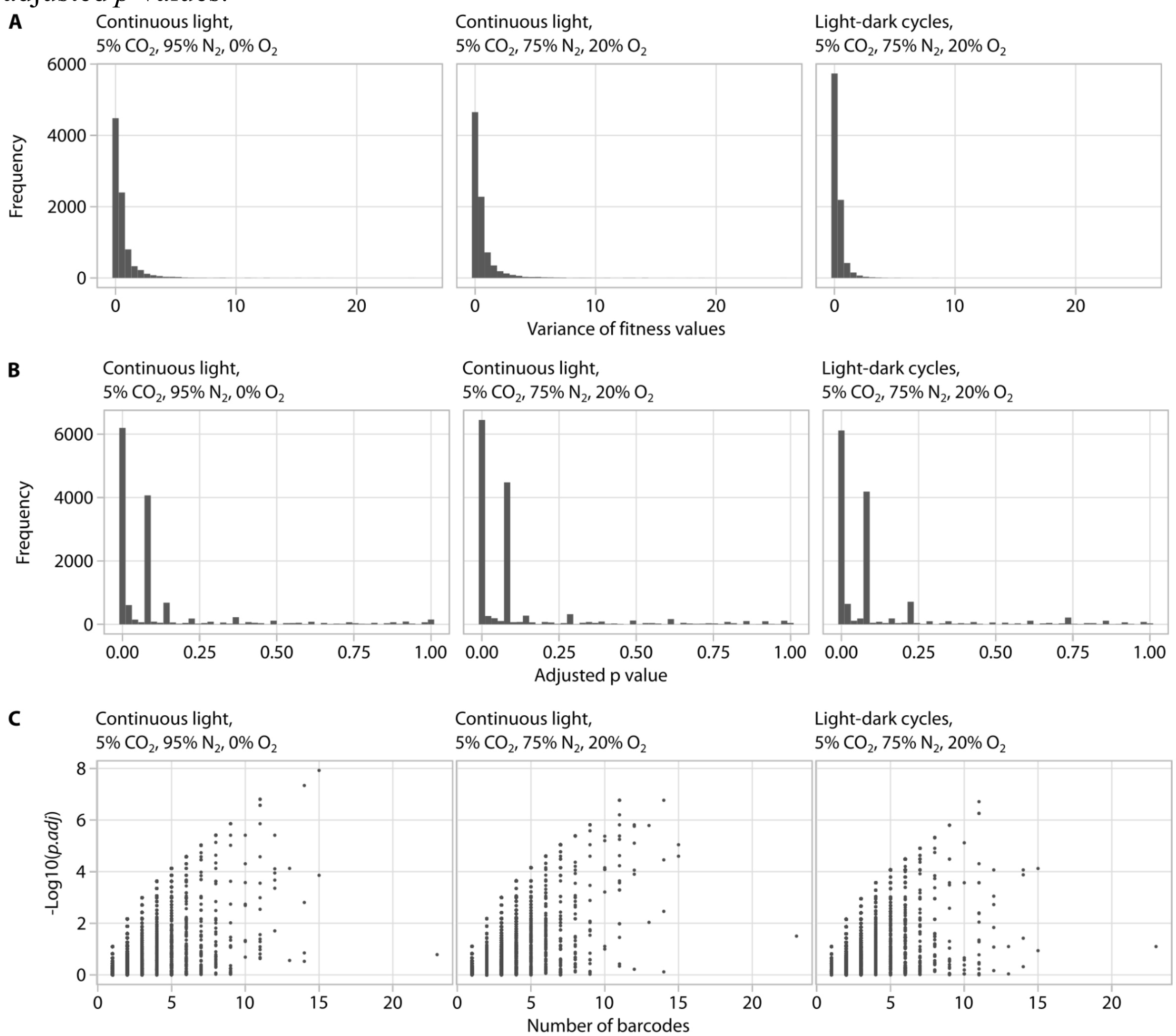

Supp. Fig. 5. Volcano plots of the variants detected in the pooled growth (related to Fig. 1) The horizontal dotted line indicates a cut-off of *p.adj*=0.05 and the vertical dotted lines cut-offs of a normalized weighted mean fitness of 0.9 and 1.1. Points in the upper left and upper right quadrant delineated by these dotted lines are colored green and red, respectively.

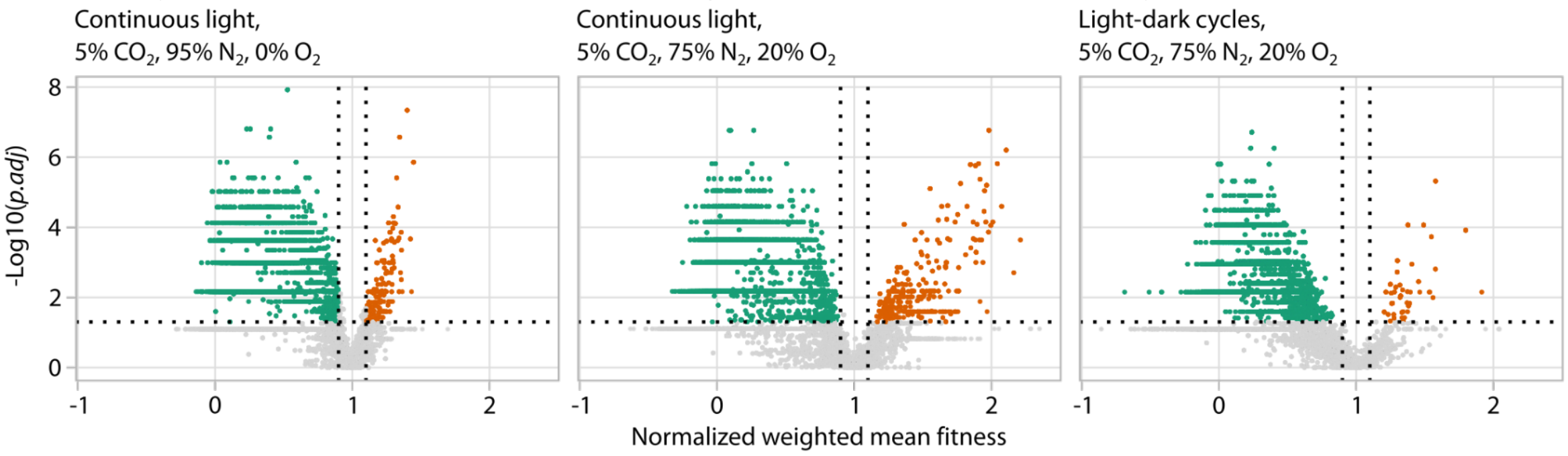

Supp. Fig. 6 (**A**) Comparison of the relative solvent accessibility of residues and the average normalized fitness score for all exchanges at that position. The dashed line gives the result of a linear regression between both variables. (**B**) Comparison of the conservation at different residue positions as determined by the EVcouplings web server and the average normalized fitness score. The dashed line gives the result of a linear regression between both variables. For both panels, Pearson's *r* is given as a small inset, *p=< 2.2e-16* for all comparisons.

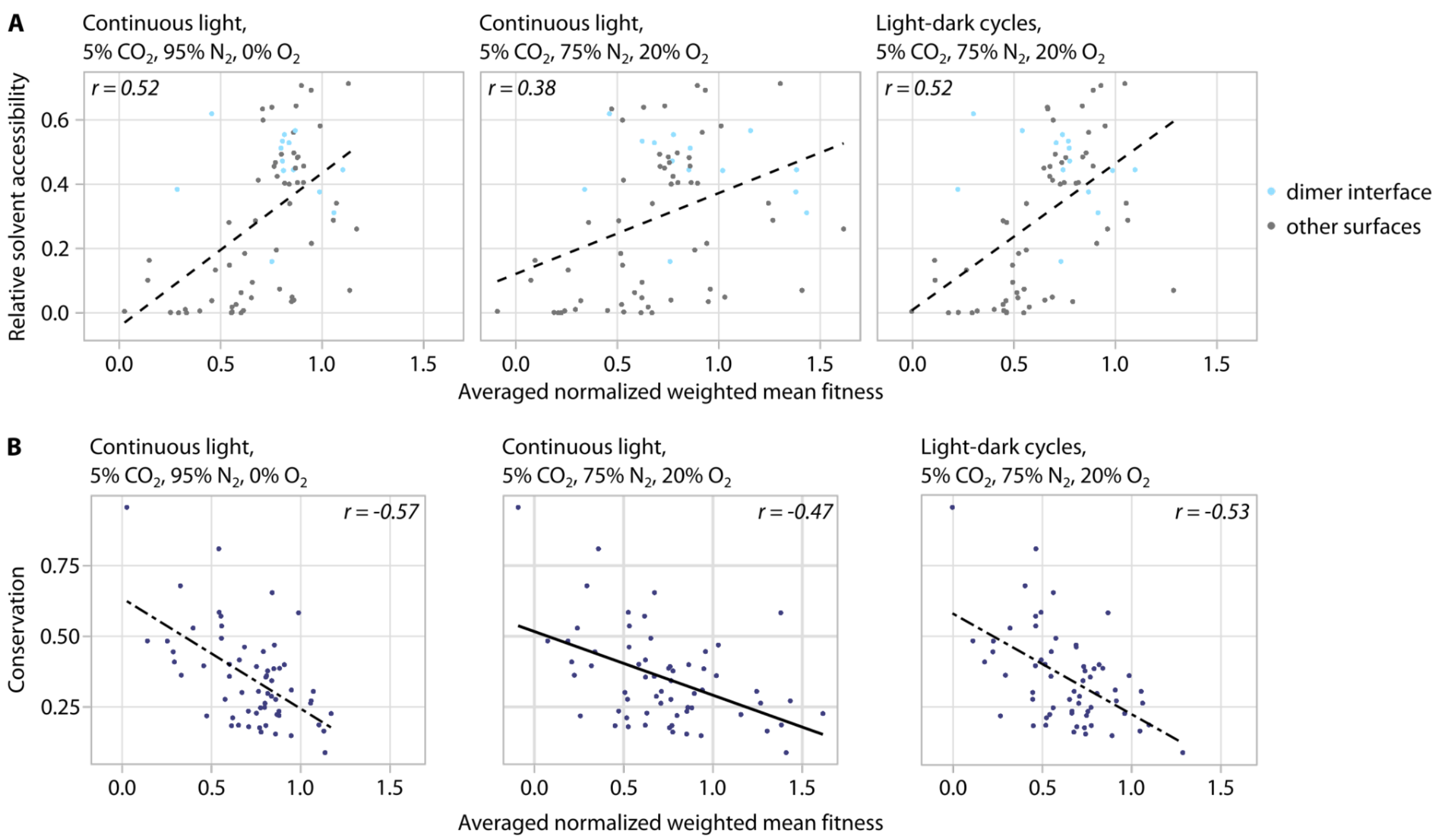

Supp. Fig. 7. (**A**) Protein structure of the CbbM Enzyme showing the residues G396-I414 (Dark blue) and tunnels (blue and brown) running from the surface to the active site. (**B**) Residues V323 (pink) and W114 (green) constrain tunnels between the surface (brown) and the active sites (blue). The magnesium ion in the active site is visualized by a red sphere. (**C**). Residue W114 at the dimer interface constricts a tunnel spanning the entire enzyme, connecting the two active sites (blue). (**D**) Mutation W144K opening up the tunnels connecting the two active sites. Active sites are marked by the orange K200 residue in panel C and D.

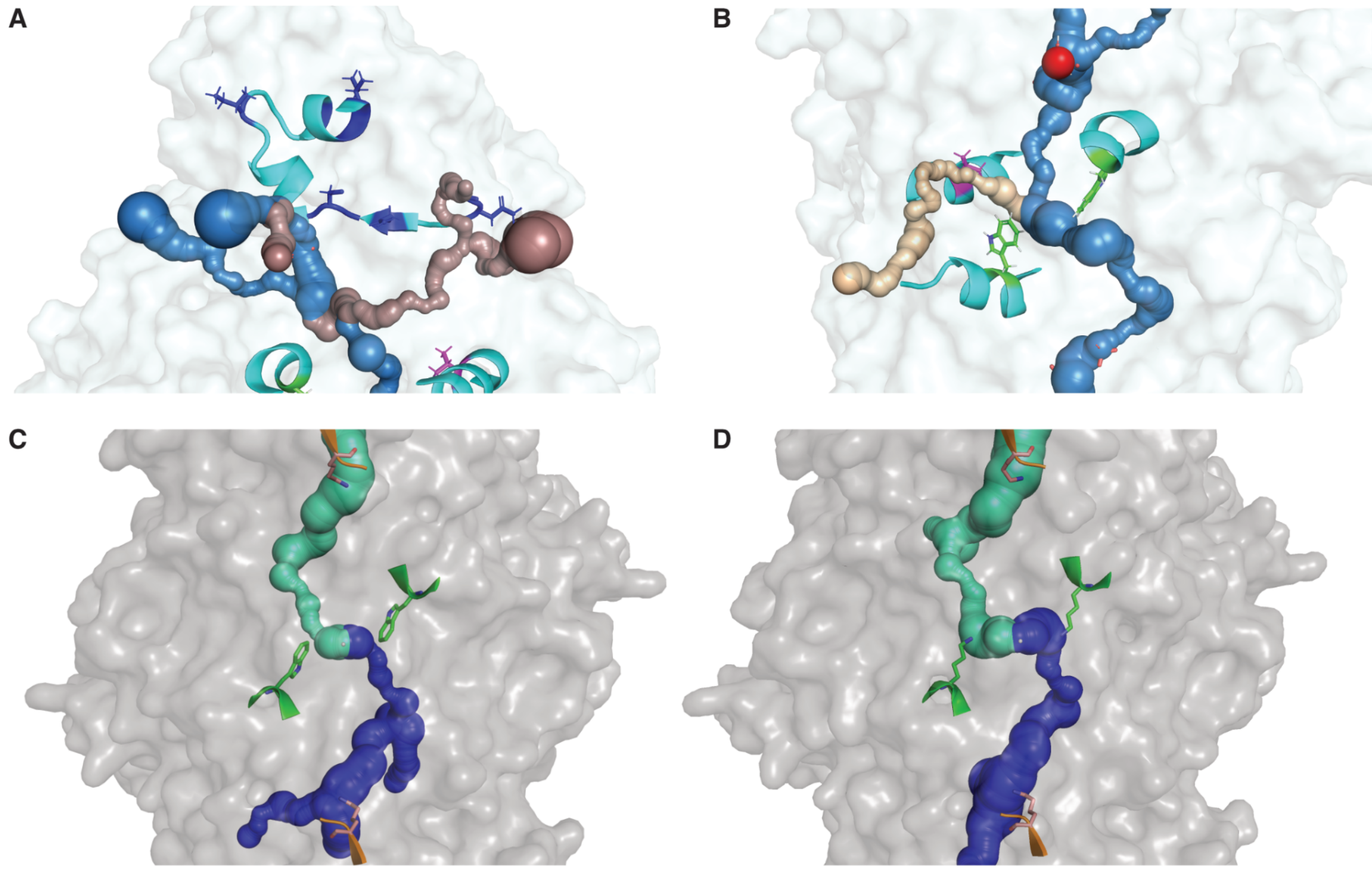

Supp. Fig. 8. Abundance of CbbM protein in soluble phase of selected variants using western blot (**A**) and load control via SDS-PAGE (**B**). Each row, starting with the reference ladder, is one gel with one sample representing triplicates. The first column to the left, for each group, is a protein ladder displaying the size of nearby markers. (**C**) Abundance of each CbbM variant in *Synechocystis* measured by LC/MS.

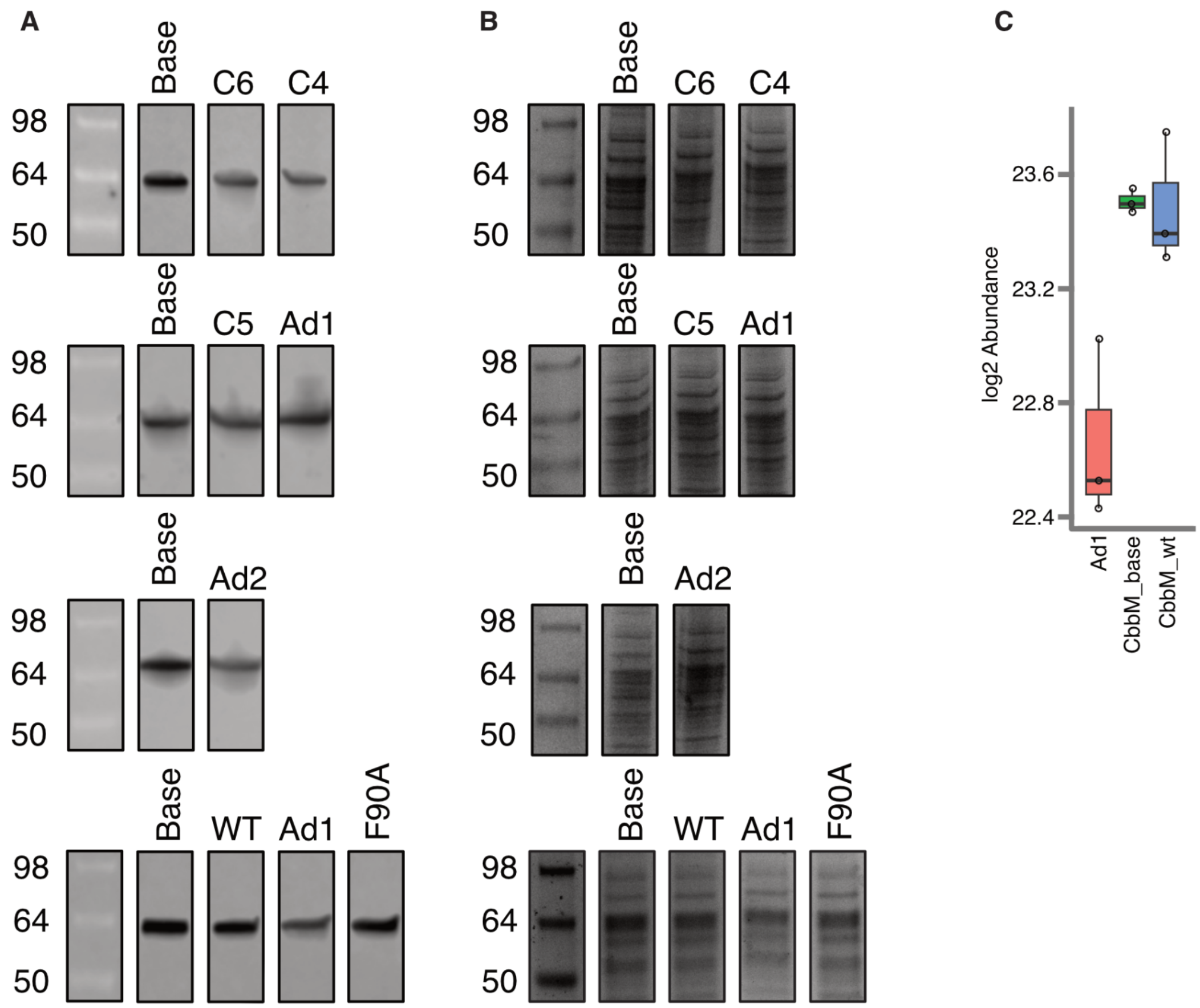

Supp. Fig. 9 Correlation of zero-shot predictors (related to Fig. 3). (**A**) EVcouplings and DeepSequence (Pearson's $r_{sat}=0.80$, $p=< 2.2e\text{-}16$ for saturational library and $r_{comb}=0.59$, $p=< 2.2e\text{-}16$ for combinatorial library) (**B**) EVcouplings and MSA Transformer (ensemble) (Pearson's $r_{sat}=0.73$, $p=< 2.2e\text{-}16$ for saturational library and $r_{comb}=0.59$, $p=< 2.2e\text{-}16$ for combinatorial library)

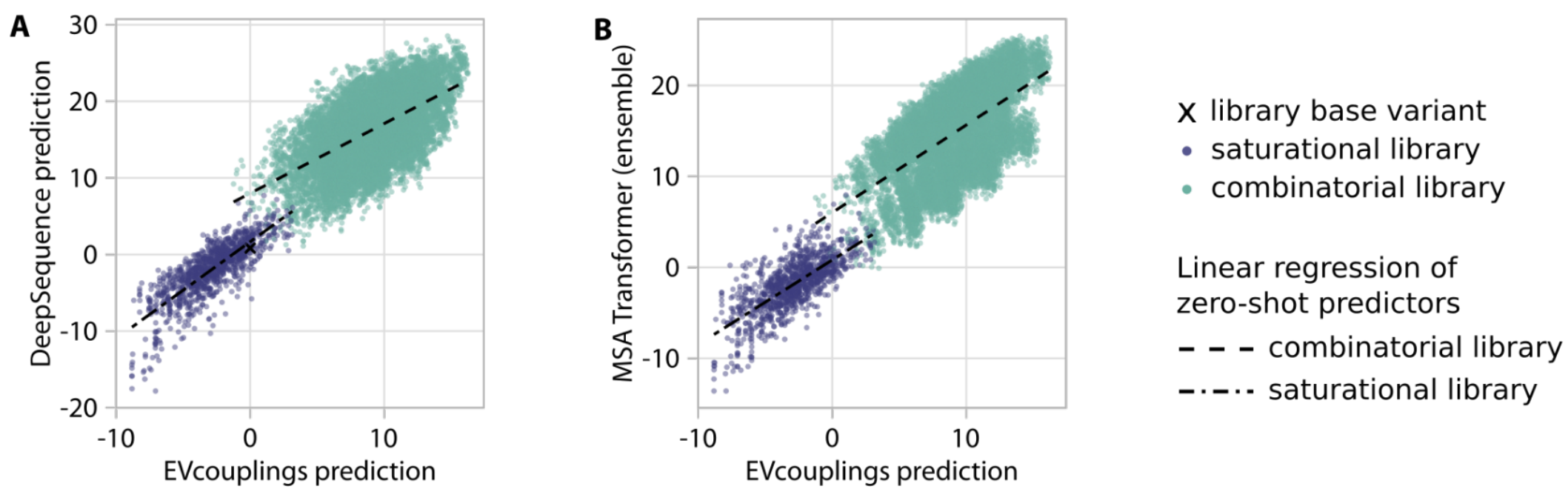

Supp. Fig. 10 Zero-shot predictions (related to Fig. 3). (**A**) EVcouplings (**B**) DeepSequence (**C**) MSA Transformer (ensemble). In all panels: r_{comb} gives the Pearson correlation between the zero-shot predictor with the experimentally measured values of the combinatorial library, r_{sat} with the experimentally measured values for the saturation library. p=< *2.2e-16* for all comparisons.

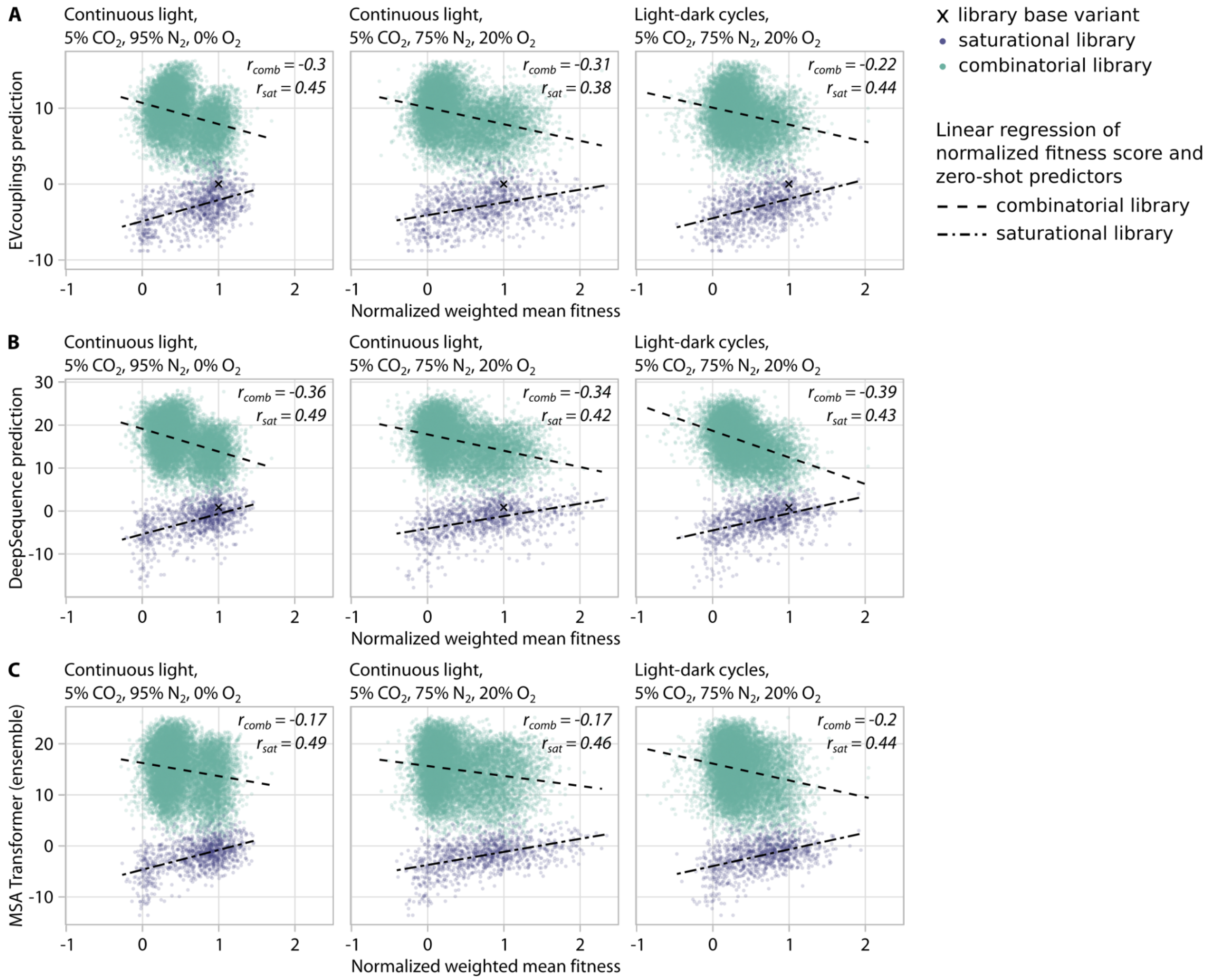

Supp. Fig 11 Zero-shot predictions when excluding V323 exchanges (related to Fig. 3). Correlation of experimental fitness of combinatorial mutants of CbbM_base and prediction of fitness using three zero-shot predictors, when fitness data from V323 exchanges are excluded. Pearson's *r* is given for all comparisons as a small inset. Corresponding *p* values are given in the order of: "continuous light, 95% N_2; continuous light, 20% O_2; light-dark cycles". (**A**) EVcouplings (*p < 2.2e-16; p < 2.2e-16; p < 2.2e-16*) (**B**) DeepSequence (*p=9.842e-09; p=0.2304; p < 2.2e-16*) and (**C**) MSA Transformer (*p=0.02245; p=7.656e-07; p=4.171e-12*).

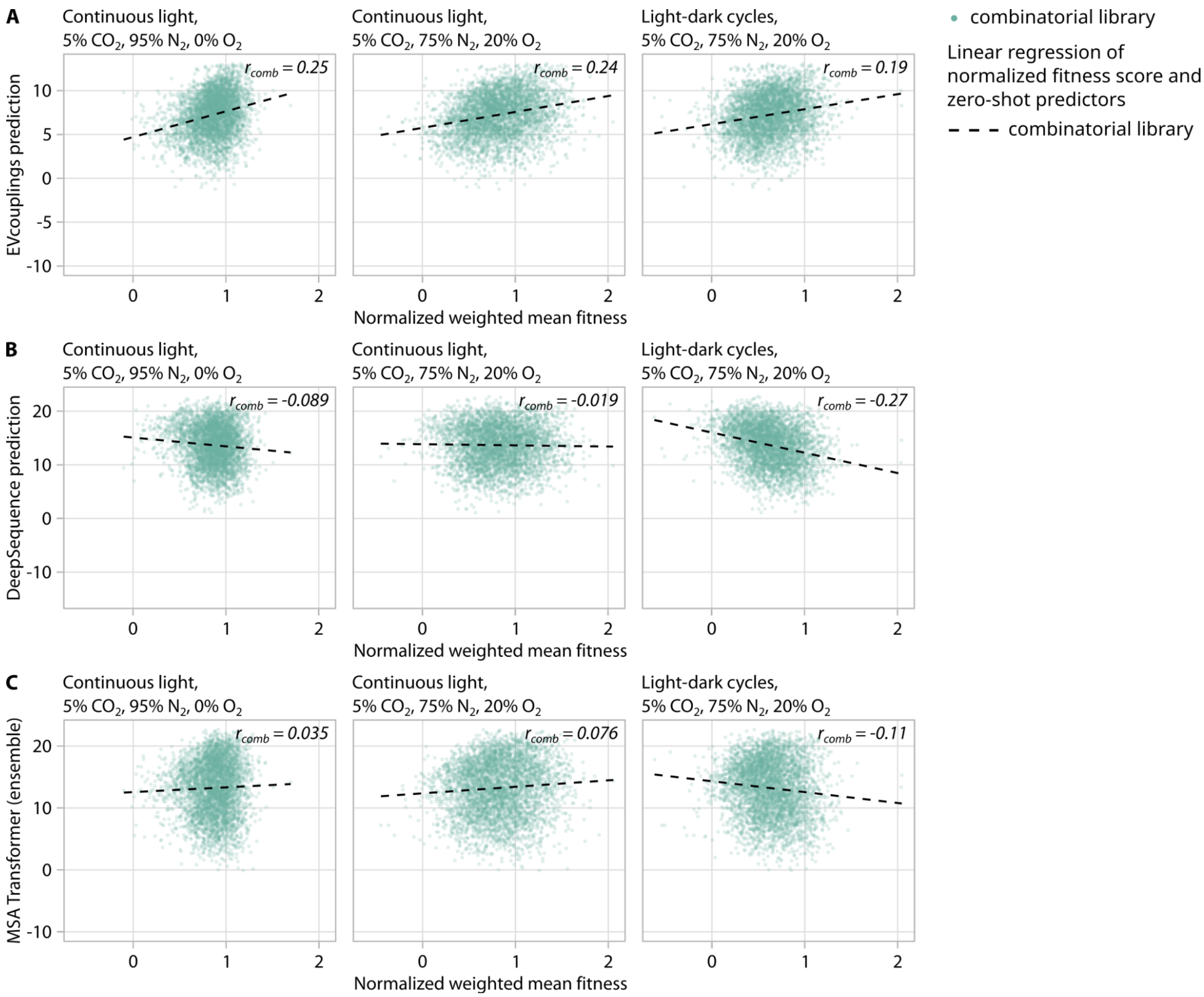

Supp. Fig. 12. Correlation of experimental fitness of combinatorial mutants of CbbM_base and prediction of fitness using the additive model. The small inserts give Pearson's *r*, for all three panels *p=< 2.2e-16*.

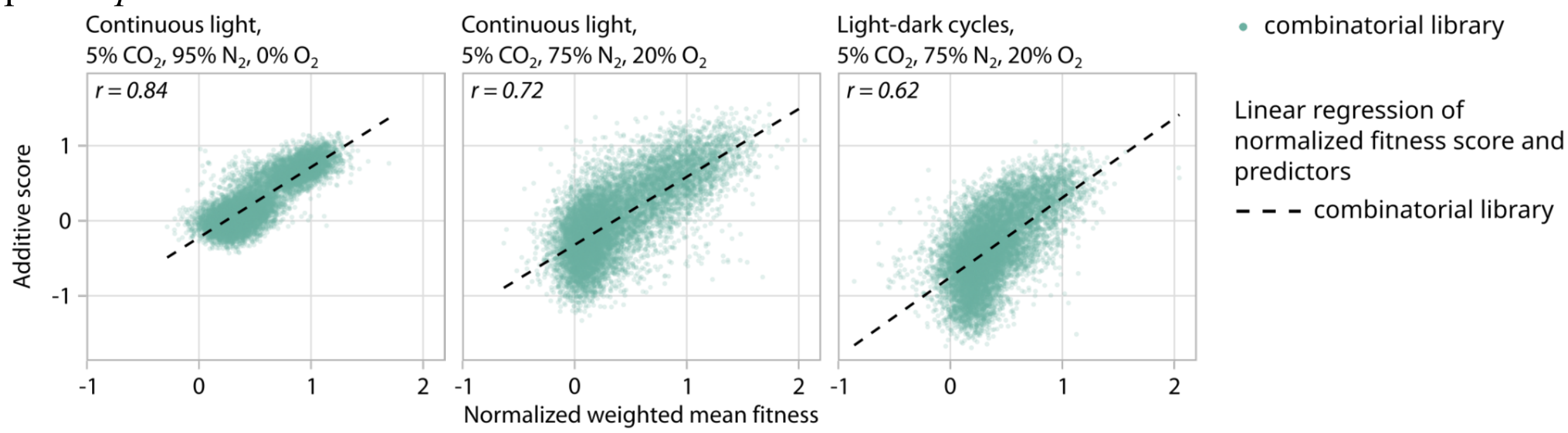

Supp. Fig. 13. Fitness predictions using MoCHI (**A**) Correlation of experimental fitness of CbbM_base and prediction of fitness using MoCHI, which was trained on single-site exchange fitness data. (**B**) Comparison of MoCHI predictions to the additive model. Pearson's *r* is given as an insert in the plots for the comparison with the combinatorial library (r_{comb}) and the saturation library (r_{sat}). The corresponding *p* values are: (A) $p < 2.2e\text{-}16$ for all r_{sat}; $p < 2.2e\text{-}16$, $p=4.166e\text{-}10$, $p < 2.2e\text{-}16$; (B) $p < 2.2e\text{-}16$ for all panels.

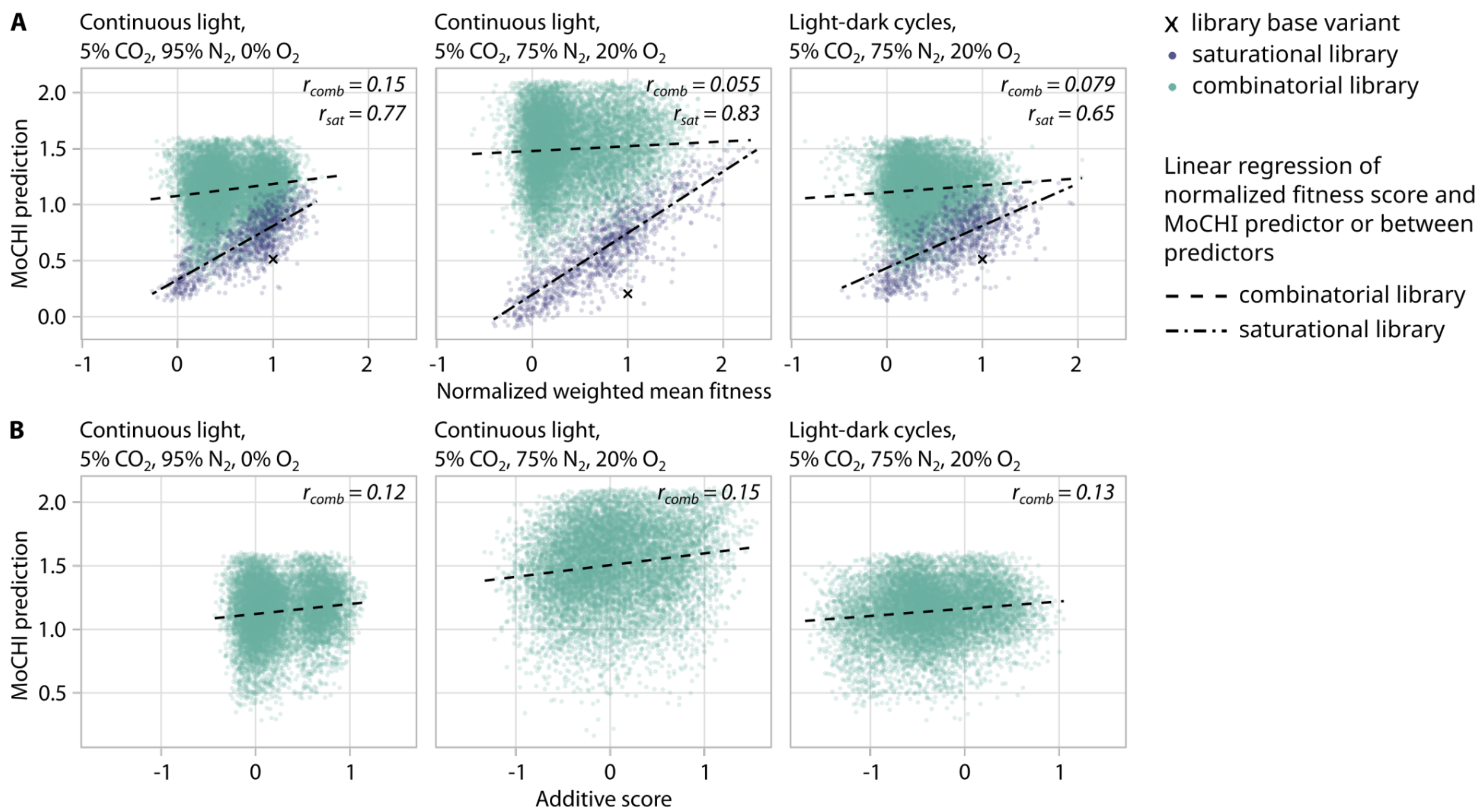

Supp. Fig. 14. Correlation of experimental fitness of combinatorial mutants of CbbM_base and prediction of fitness using ProteinNPT, which was trained on single-site exchange fitness data. Predictions are shown including (**A**) and excluding (**B**) fitness from V323 exchanges. (**C**) Comparison of ProteinNPT predictions to the additive model. Pearson's *r* is given as an insert in the plots. The corresponding *p* values are: (A) for all panels $p < 2.2e\text{-}16$; (B) $p=0.6057$, $p < 2.2e\text{-}16$, $p < 2.2e\text{-}16$; (C) for all panels $p < 2.2e\text{-}16$.

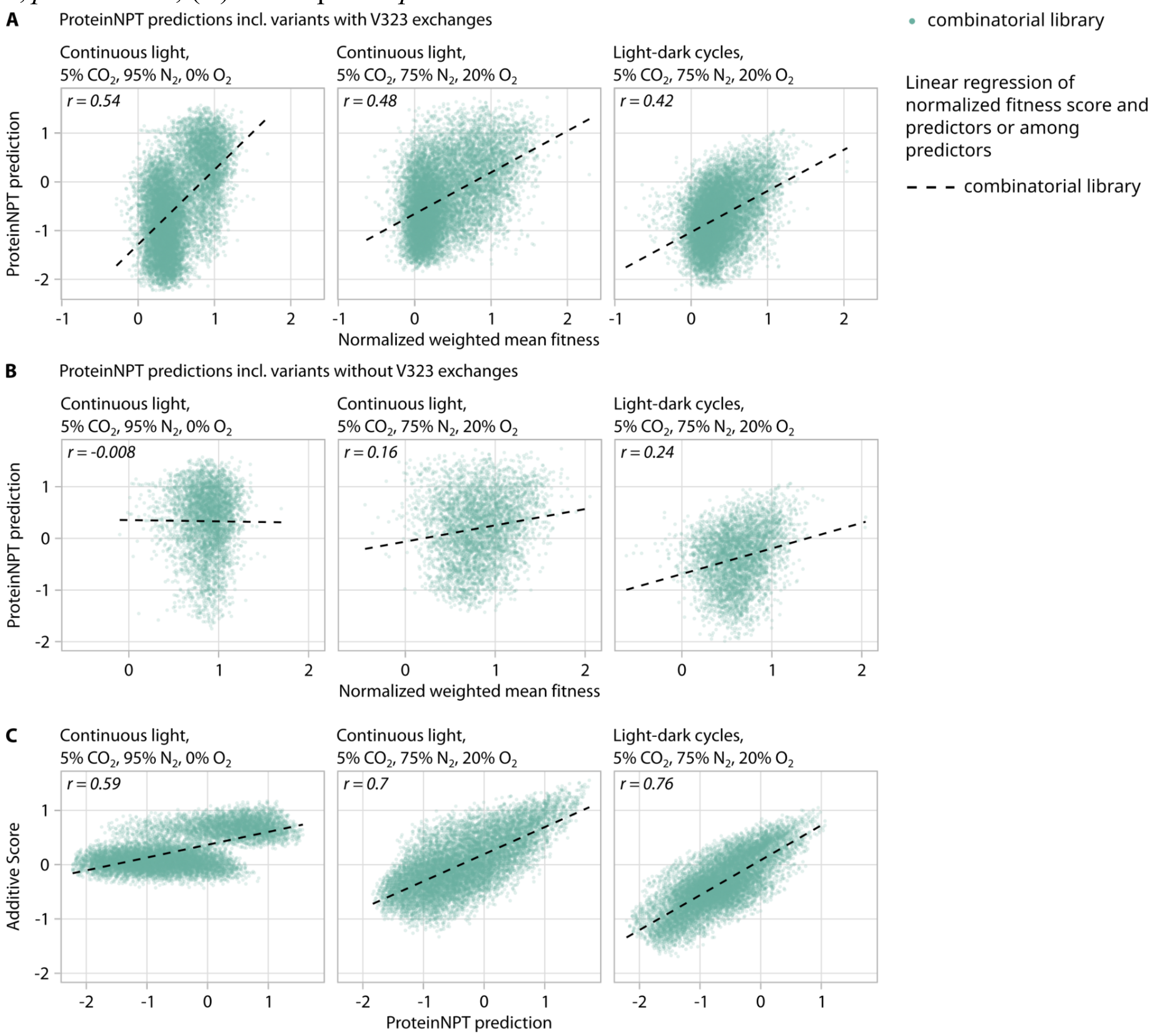

Supp. Fig. 15. Growth of selected mutants and Base variant in low CO2 and in nitrogen. (A) Growth at 1 % CO2 in air, 50 µmol photons m-2 s-1 and 30 °C. (**B**) Growth of variants in multicultivator at 5 % CO2, 95 % N2, at 50 µmol photons m-2 s-1 and 30 °C. For both cultivations, starting OD_{730} was 0.05, and the growth test was started ~ 96 h after aTc addition.

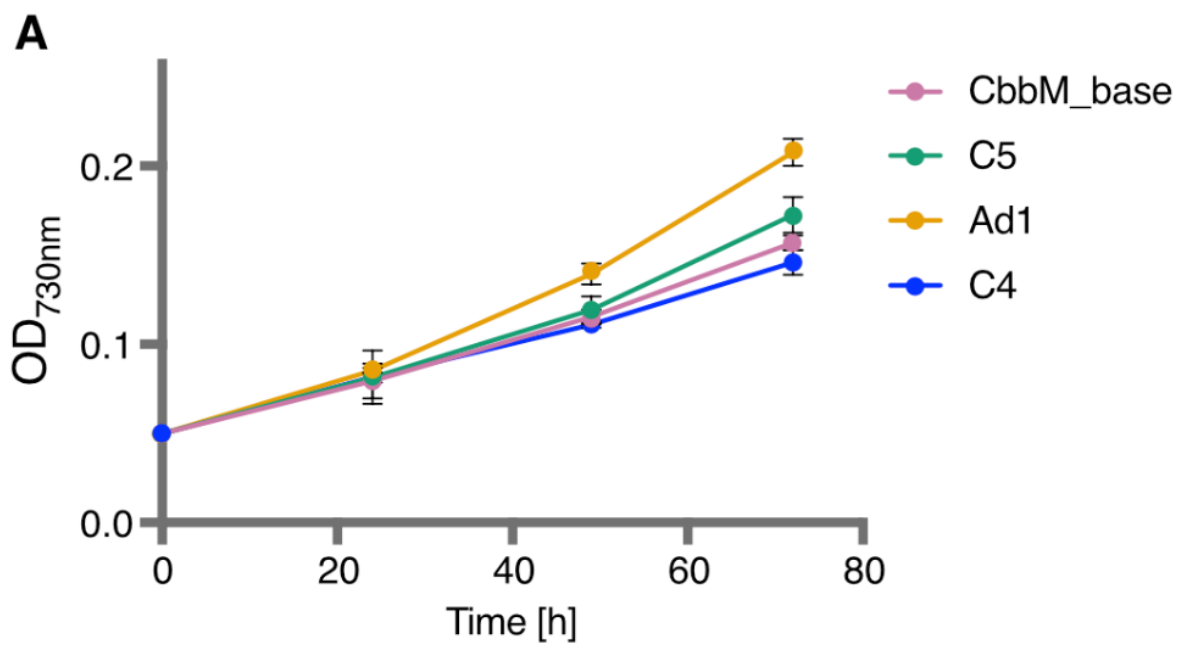

Supp. Fig. 16. Thermal melting curves of selected CbbM variants expressed in and purified from *E. coli* as determined by nanoDSF.

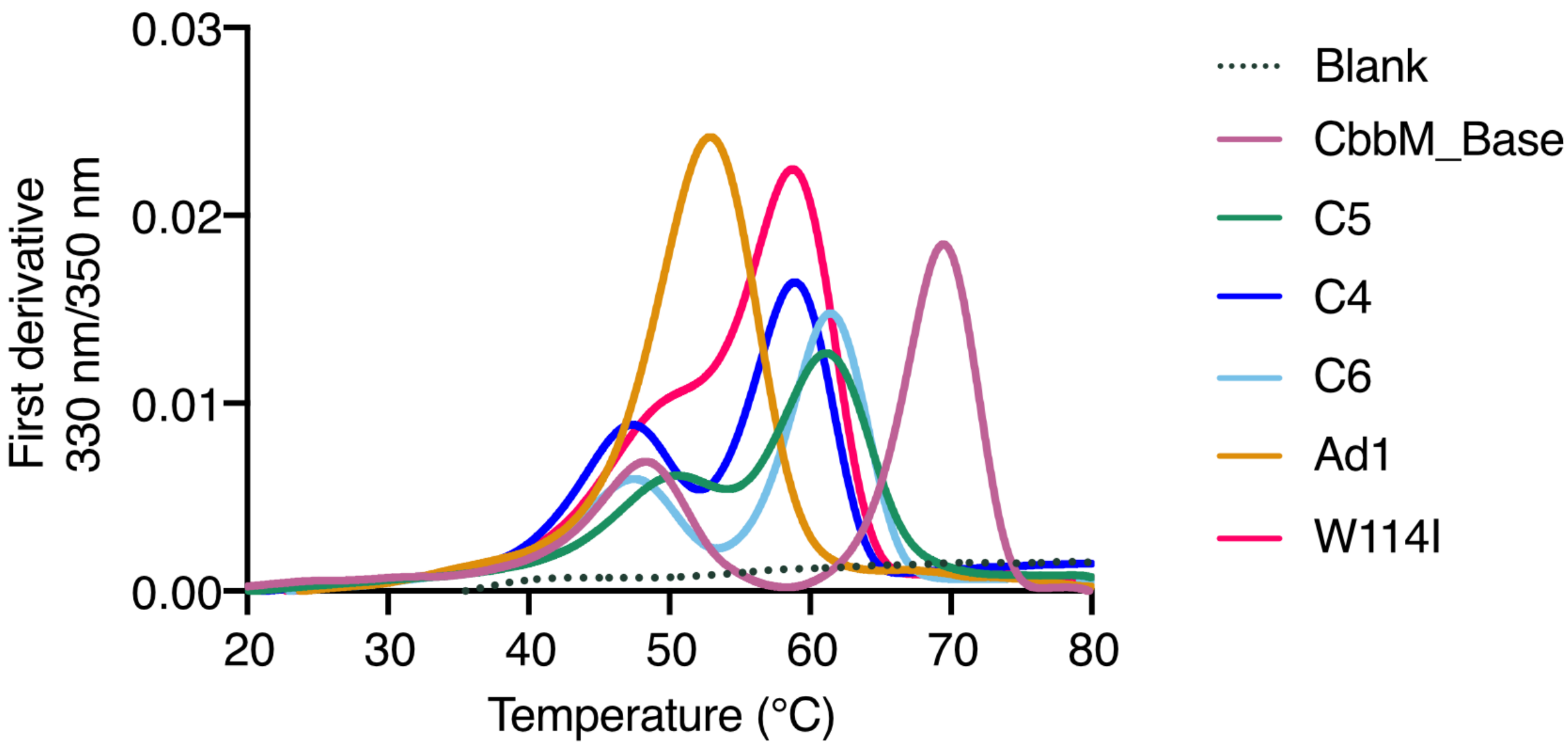

Supp. Fig. 17. Histogram with data of the size of the protein from Mass photometry of Base variant (blue) and Ad1 (yellow).

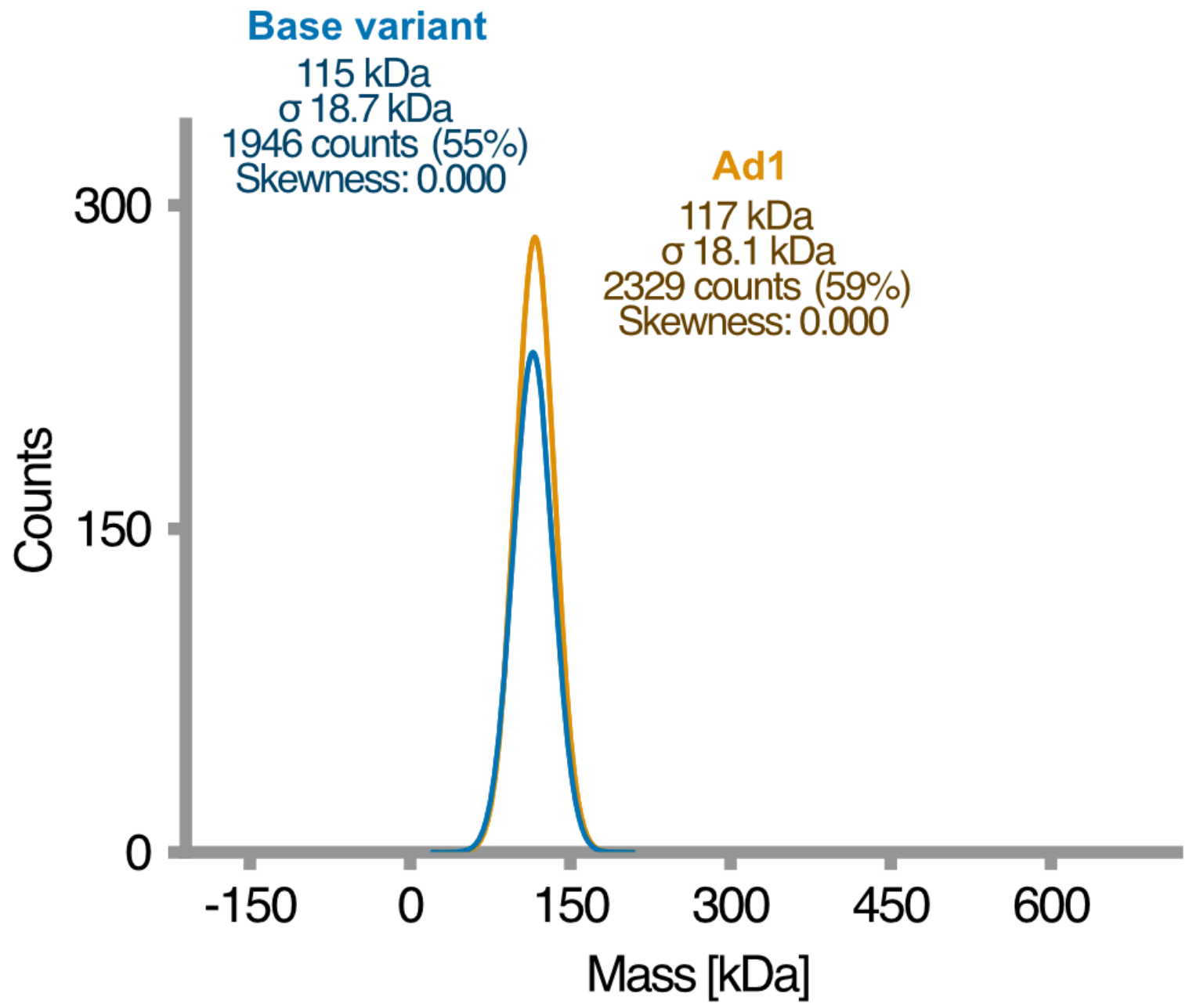

Supp. Fig. 18. Volcano plots of proteomics data of *Synechocystis* growing with (**A**) Ad1 variant (**A**) and the (**B**) CbbM_wt compared to *Synechocystis* growing with the CbbM_base. Samples were taken 100 h after induction with aTc which represses native RbcLS. The strains were grown in continuous light and a gas feed of 5% CO_2, 75% N_2, 20% O_2. Proteins are labeled with their gene name in case they were significantly changed.

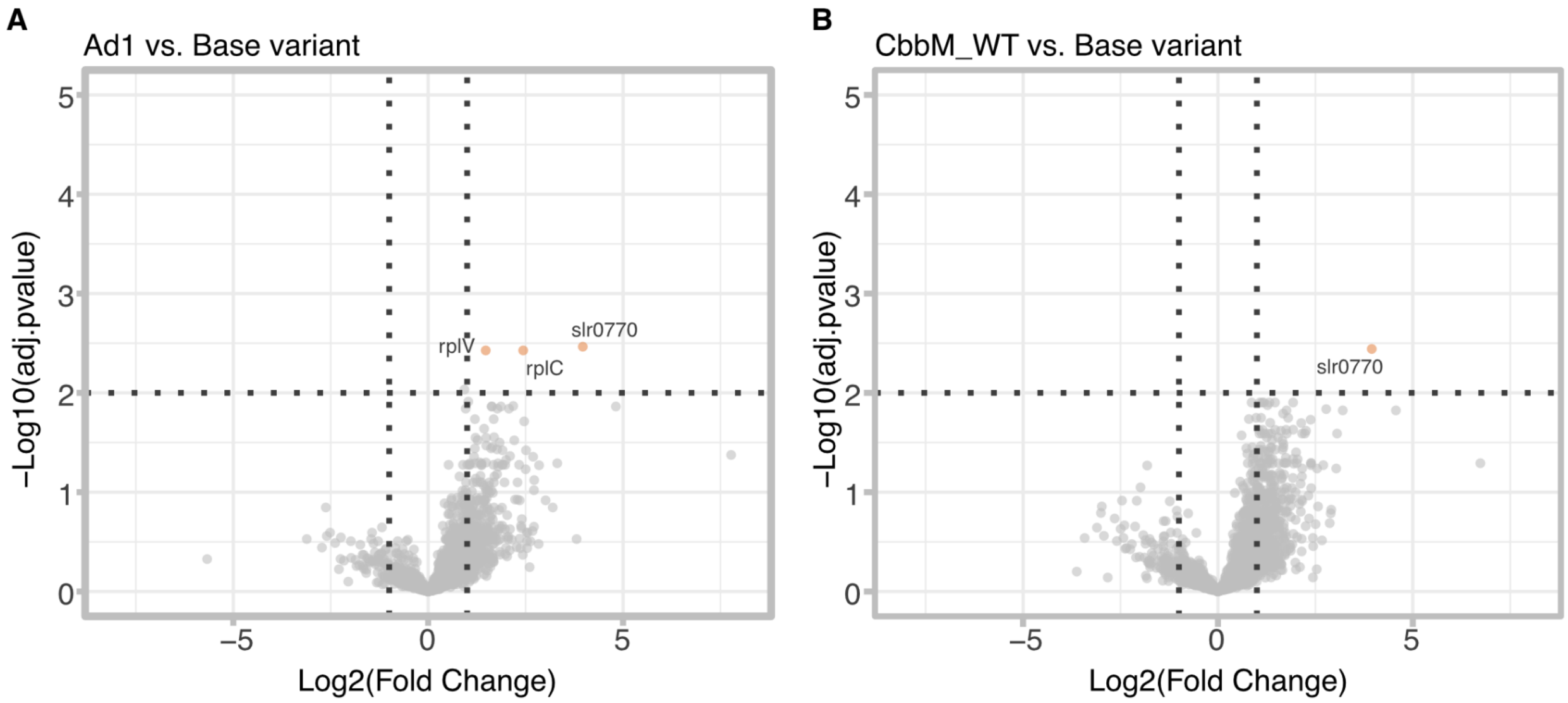

Supp. Fig. 19. Oxygen evolution rates in µmol O_2 µg Chl^{-1},h^{-1} for selected strains measured using a Clark-type electrode. For CbbM containing strains, measurements are started at ~100 hours after induction with 2 µg ml^{-1} aTc to repress RbcLS. For $CRISPRi_{rbcLSX}$ strain, no aTc was added. Strains were grown at 50 µmol photons m-2 s-1, 30 °C and 5% CO_2, 75% N_2, 20% O_2 **.**The oxygen levels were measured in darkness for the first five minutes. At 5 min the light was turned on at an intensity of 100 µmol photons m-2 s-1. At 10 minutes 20 µM DCMU was added to stop water splitting and O_2 production. **(A)** Oxygen consumption rate during the first five minutes in darkness. **(B) Net** Oxygen evolution rate during the light period **(C)** Oxygen consumption rate after the addition of 20 µM DCMU. (D) Full oxygen evolution procedure.

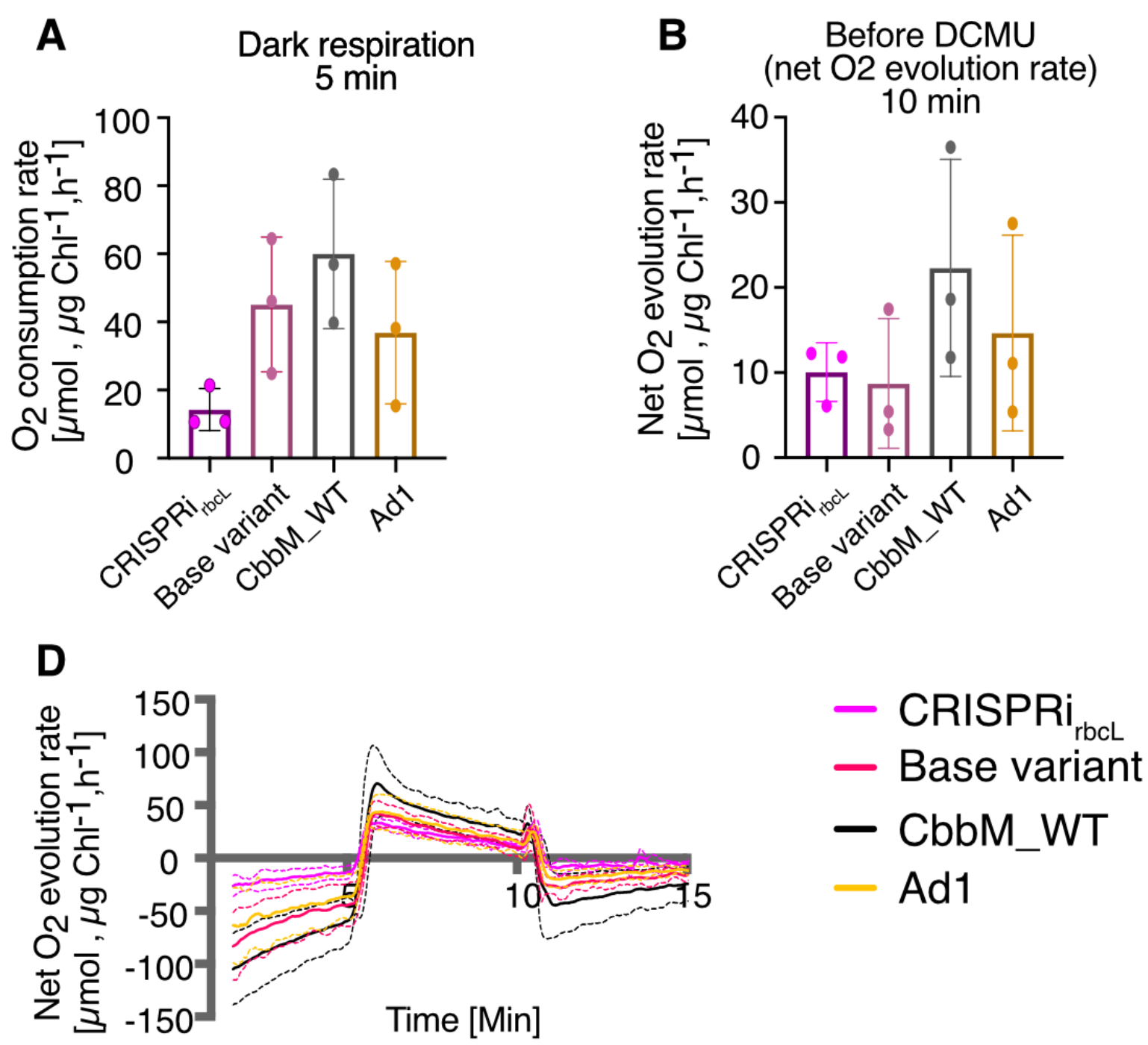

Supp. Fig. 20. Flow chart illustrating the computational and experimental steps leading from wild-type CbbM (CbbM_wt) to the evolved variants. The chart includes experiments performed to characterize the evolved variants.

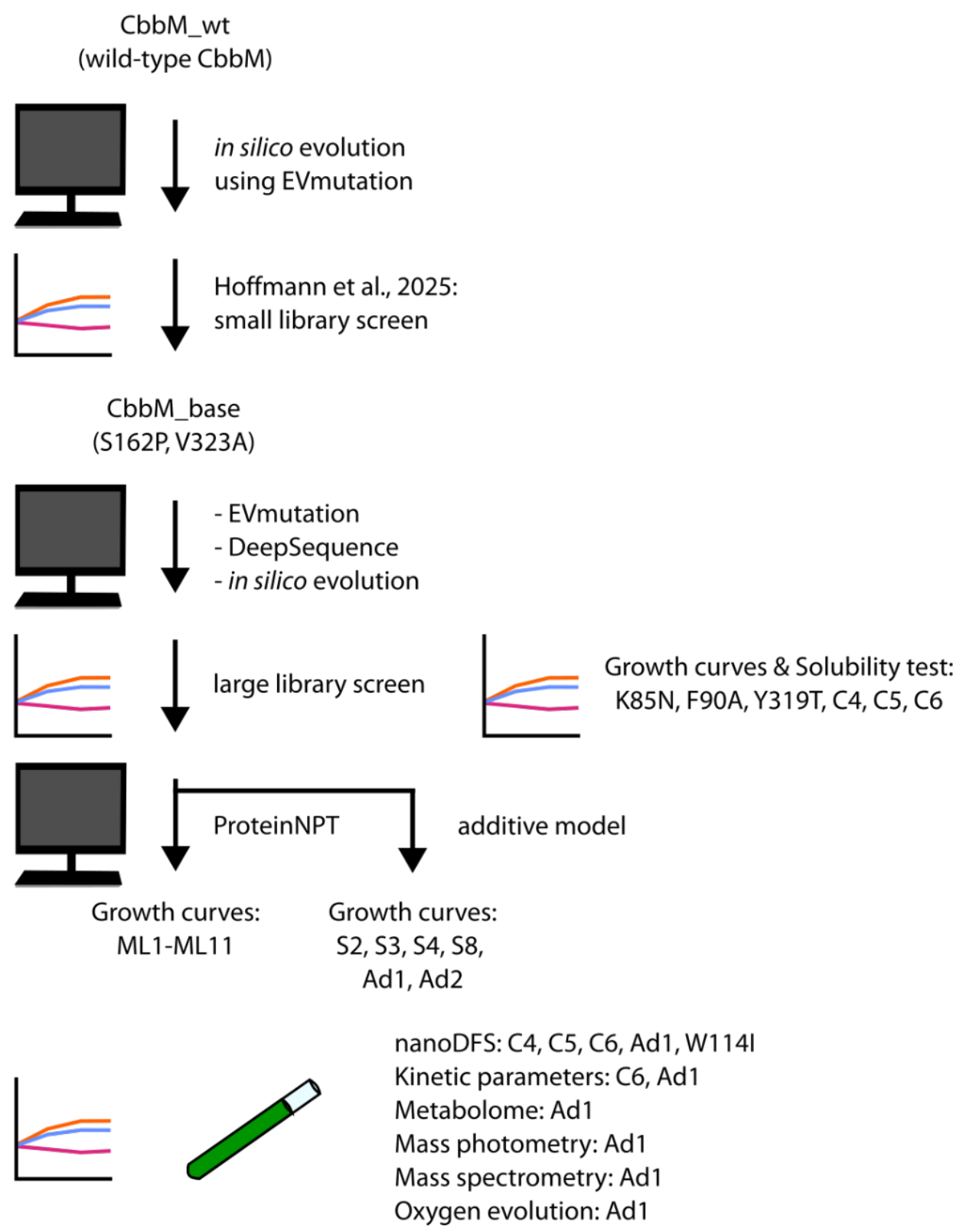

Supp. Table 1: All 71 positions included in the deep mutational sequencing (DMS) part of the library. Amino acid positions are given for the protein sequence without N-terminal Strep tag (column "no N-Strep") and with N-terminal Strep tag (column "with N-Strep"). A small x indicates if an exchange was included based on EVmutation (column "EV") or DeepSequence (column "DS") predictions. For potentially acetylated lysines, the amino acids in close proximity which triggered the inclusion in the library are given (column "K"). Several amino acid positions were included for various other reasons (column "various"). Note that EVmutation and DeepSequence predictions for all exchanges are available in Supp. Data 1.

no N-Strep	with N-Strep	EV	DS	K	various
K13	K27			E	
K19	K33			N	
K29	K43		x		
I31	I45	x	x		
T77	T91		x		
V84	V98	x	x		
K85	K99	x	x		
F90	F104	x	x		
V92	V106	x			
N109	N123		x		
H112	H126	x	x		
W114	W128	x			
S115	S129		x		
L118	L132		x		
H127	H141	x	x		
Q128	Q142	x			

L136	L150	x	x		
M140	M154	x	x		
V142	V156		x		
K144	K158			R, F	
A150	A164		x		
N154	N168	x	x		
K200	K214				negative control
E214	E228		x		
V215	V229	x	x		
M217	M231	x			
K219	K233	x			
A230	A244	x			
Q233	Q247		x		
Y245	Y259	x	x		
K247	K261		x		
H251	H265	x	x		
D254	D268	x			
F255	F269	x	x		
V256	V270	x	x		
A261	A275	x			
K262	K276		x		

A281	A295		x		
E289	E303		x		
Y308	Y322		x		
K309	K323			Y, S	
M312	M326		x		
M314	M328		x		
D315	D329		x		
Y319	Y333		x		
L322	L336		x		
V323	V337	x			
M326	M340		x		
M331	M345	x	x		
H344	H358		x		
G345	G359		x		
K346	K360	x	x		
Y365	Y379		x		
K367	K381		x	Q	
Q392	Q406	x	x		
G397	G411		x		
N398	N412				literature
N401	N415				literature

C403	C417		x	
I411	I425		x	
A415	A429		x	
G418	G432	x	x	
I419	I433	x		
K429	K443			S, W
S430	S444	x		
S432	S446	x	x	
K439	K453			T
K442	K456			E
K453	K467			D
K457	K471			D
K465	K479			E

Supp. Table 2: Composition of the library used for growth competition. The number of expected variants according to the library design are compared to the number of variants and number of barcodes observed in the Synechocystis mutant pool used for experiments. Base variant: CbbM(WT) with exchanges A230E and G422R, variant underlying the complete library design. "Spontaneous" describes variants which occurred spontaneously. The count of spontaneous variants were not included in the total of the number of observed variants. If spontaneous variants are included, the total number of observed variants is 13,966.

Library part	Expected number variants	Observed number variants (% of expected)	Number barcodes (average per variant)
Base variant	1	1 (100.0)	30 (30.0)
Combinatorial higher-order variants	16,178	12,708 (78.6)	25,793 (2.0)
Saturational	1,349	1,223 (90.7)	4,566 (3.7)
Spontaneous	-	34 (-)	69 (2.0)
Total	17,528	13,932 (79.5)	30,459 (2.2)

Supp. Table 3: Proteomics data comparing samples taken at generation 4 or 8 compared samples taken at generation 0, i.e. before addition of aTc. Log 2 fold changes (Log2FC) are marked in bold and color if the corresponding adjusted p value (adj. p) is below 0.01. Data for a subset of proteins is shown. The complete data set is available online.

			Generation 4/ Generation 2		Generation 8/ Generation 2	
	Locus tag	Gene name	Log2FC	adj. p	Log2FC	adj. p
dCas9	*NA*	*dCas9*	**2.51**	2.6E-04	**2.46**	2.9E-04
Rubisco	*slr0009*	*rbcL*	**-3.81**	2.97E-04	**-4.97**	7.06E-05
Rubisco	*slr0012*	*rbcS*	**-3.77**	3.54E-04	**-5.28**	6.87E-05
Carbonic anhydrase	*slr1347*	*ccaA*	**-1.27**	1.98E-04	**-1.54**	7.06E-05
Carboxysome	*sll1028*	*ccmK2*	1.53	6.62E-02	1.30	9.61E-02
Carboxysome	*sll1029*	*ccmK1*	**1.68**	9.49E-04	**1.80**	5.63E-04
Carboxysome	*sll1030*	*ccmL*	2.21	1.49E-02	**3.21**	2.79E-03
Carboxysome	*sll1031*	*ccmM*	**-1.87**	1.28E-04	**-2.32**	5.02E-05
NdhR regulon	*sll1594*	*ndhR*	**1.01**	1.60E-03	**0.93**	2.30E-03
NdhR regulon	*sll1732*	*sll1732*	1.00	4.10E-01	2.06	9.24E-02
NdhR regulon	*sll1733*	*ndhD3*	**3.74**	2.88E-05	**3.88**	2.59E-05
NdhR regulon	*sll1734*	*cupA*	**3.18**	1.90E-05	**3.57**	6.87E-06
NdhR regulon	*sll1735*	*sll1735*	**1.84**	1.65E-03	**1.66**	2.57E-03
NdhR regulon	*slr1512*	*sbtA*	**3.49**	1.28E-05	**3.99**	4.14E-06
NdhR regulon	*slr1513*	*sbtB*	**3.09**	3.44E-03	**3.38**	2.26E-03
NdhR regulon	*slr2011*	*slr2011*	-0.44	7.21E-01	-0.15	8.92E-01

Supp. Table 4: Number of variants with a higher fitness value than the library base variant in (**A**) 5% CO2, 95% N2, 0% O2, continuous light (**B**) 5% CO2, 75% N2, 20% O2, continuous light cultivation conditions. Using the Wilcoxon-Rank test, p values were calculated by comparing the fitness values of barcodes associated with a specific variant and fitness values of barcodes associated with the base variant. These p values were adjusted for multiple testing using Benjamini-Hochberg. Percentages given after the total number of variants are referring to the total number of observed variants, i.e. the 13,966 variants that include spontaneously occurring variants.

(**A**) Growth condition: 5% CO2, 95% N2, 0% O2, continuous light

Library part	Variants with a higher fitness value than the base variant (percentage of respective part of library)	Variants with a higher fitness value and adjusted $p < 0.05$ (percentage of respective part of library)
Combinatorial	1,059 (8.3)	93 (0.7)
Saturational	291 (23.8)	87 (7.1)
Spontaneous	1 (2.9)	0 (0.0)
Total	1,351 (9.7)	180 (1.3)

(**B**) Growth condition: 5% CO2, 75% N2, 20% O2, continuous light

Library part	Variants with a higher fitness value than the base variant (percentage of respective part of library)	Variants with a higher fitness value and adjusted $p < 0.05$ (percentage of respective part of library)
Combinatorial	1,248 (9.8)	144 (1.1)
Saturational	333 (27.2)	135 (11.0)
Spontaneous	3 (8.8)	0 (0.0)
Total	1,584 (11.3)	280 (2.0)

Supp. Table 5: Variants of CbbM_base with the highest normalized fitness score, which are better than the base variant and which were significantly better than the base variant (p.adj < 0.05) under all three conditions. "Product normed values" gives the product of the normalized fitness scores under all three conditions. Variants underlying the combinations used for ProteinNPT predictions are marked by an asterisk (*). Note that variant numbering includes a 14-residue N-terminal Strep tag (e.g. W128I is W114I when excluding Strep tag).

Variant	Normalized fitness score			adjusted p value compared to library base variant			
	5% CO2, 95% N2, 0% O2	5% CO2, 75% N2, 20% O2	LD, 5% CO2, 75% N2, 20% O2	5% CO2, 95% N2, 0% O2	5% CO2, 75% N2, 20% O2	LD, 5% CO2, 75% N2, 20% O2	Product normed values
H126N*	1.23	1.97	1.92	2.52E-02	2.54E-02	7.02E-03	4.64
H126M	1.23	1.96	1.80	8.49E-03	7.05E-05	1.21E-04	4.33
W128I*	1.42	1.98	1.39	2.14E-04	1.37E-04	1.48E-02	3.91
K360I*	1.40	1.98	1.38	4.58E-08	1.72E-07	8.57E-05	3.82
K360M	1.30	1.75	1.55	7.49E-05	4.32E-05	1.86E-04	3.53
Q142D*	1.29	1.89	1.41	6.87E-03	1.00E-03	1.12E-03	3.41
K233C*	1.22	1.81	1.45	6.48E-03	6.05E-04	3.52E-03	3.20
H358C*	1.29	1.91	1.30	2.13E-04	1.53E-06	1.88E-03	3.18
K360L	1.23	1.67	1.53	2.52E-02	2.54E-02	7.02E-03	3.14
H358L	1.31	1.88	1.26	7.80E-05	1.75E-06	1.46E-02	3.13
K99N*	1.20	1.55	1.58	2.04E-02	8.60E-03	1.55E-03	2.94
K360C	1.29	1.70	1.26	6.63E-04	2.28E-04	1.48E-02	2.76
Y333V*	1.21	1.77	1.25	2.88E-03	5.63E-06	4.21E-03	2.69
H126G	1.17	1.35	1.58	2.38E-04	6.70E-03	4.81E-06	2.50

M154E, K261A, Q406D, S446T*	1.27	1.46	1.33	1.31E-02	2.54E-02	3.78E-02	2.47
Q142E	1.12	1.58	1.37	4.63E-02	6.57E-03	6.83E-03	2.43
M345Q*	1.20	1.55	1.30	4.41E-04	7.87E-06	8.94E-04	2.42
F104V*	1.22	1.49	1.32	9.00E-04	6.05E-04	9.90E-03	2.40
V98Q*	1.20	1.59	1.23	6.85E-03	6.57E-03	3.06E-02	2.36
K261A, H265A, Q406D, S446I*	1.22	1.43	1.34	3.71E-02	2.54E-02	2.55E-02	2.34
K99T	1.17	1.35	1.28	1.59E-02	4.12E-03	3.87E-02	2.01
K233L	1.14	1.37	1.28	6.85E-03	1.87E-02	7.02E-03	2.00
M345V	1.14	1.17	1.20	4.44E-02	4.91E-02	2.60E-02	1.61

Supp. Table 6: List of all 11 selected mutants created using ProteinNPT. The mutations are on top of the base variant (e.g. also contains S162P and G422R). Note that variant numbering includes a 14-residue N-terminal Strep tag (e.g. W128I is W114I when excluding Strep tag).

Name	Mutations
ML1	F104V:H126N:Q142D:K261A:H265A:K360I:Q406D:S446I
ML2	K99N:F104V:H126N:Q142D:K261A:H265A:K360I:Q406D:S446I
ML3	V98Q:F104V:W128I:Q142D:K261A:H265A:K360I:Q406D:S446I
ML4	H126N:Q142D:K261A:H265A:Q406D:S446I
ML5	V98Q:F104V:H126N:Q142D:K261A:H265A:Y333V:M345Q:H358C:Q406D:S446I
ML6	H126N:Q142D:K261A:H265A:Y333V:Q406D:S446I
ML7	W128I:Q142D:K261A:H265A:Q406D:S446I
ML8	V98Q:F104V:H126N:Q142D:K233C:K261A:H265A:Y333V:Q406D:S446I
ML9	V98Q:F104V:H126N:Q142D:K233C:K261A:H265A:K360I:Q406D:S446I
ML10	V98Q:K99N:F104V:H126N:W128I:Q142D:K261A:H265A:H358C:K360I:Q406D:S446I
ML11	V98Q:K99N:F104V:H126N:W128I:K261A:H265A:K360I:Q406D:S446I

Supplementary Data File 1. Fitness predictions and associated data for all variants present in CbbM library.

- Tab 1: Predicted and experimentally determined fitness scores, calculated surface area, predicted dimer interface for all CbbM variants. Normalized weighted mean fitness scores are given for three conditions; CL_N2: continuous light, oxygen-free gas feed, CL_O2: continuous light, oxygen-containing gas feed, LD: light-dark cycles, oxygen-containing gas feed.
- Tab 2: Kinetic parameters of CbbM variants determined by *in vitro* assay.

Supplementary Data File 2. Quantification of selected sugar-phosphate metabolites from *Synechocystis* growing with CbbM variants.

- Tab 1: Calculated metabolite concentrations over 3-day cultivation (μmol/gDCW)
- Tab 2: Area under curve for respective elution peaks.
